# HIV-1 cell-to-cell infection of macrophages escapes type I interferon and host restriction factors, and is resistant to antiretroviral drugs

**DOI:** 10.1371/journal.ppat.1013130

**Published:** 2025-04-28

**Authors:** Marie Woottum, Sen Yan, Agathe Durringer, Léa Mézière, Lucie Bracq, Mingyu Han, Delphine Ndiaye-Lobry, Julie Chaumeil, Jean-Christophe Pagès, Serge Benichou

**Affiliations:** 1 Institut Cochin, Inserm U1016, Paris, France; 2 CNRS, UMR8104, Paris, France; 3 Université Paris Cité, Paris, France; 4 Institut RESTORE, Université Toulouse 3, CNRS U-5070, EFS, ENVT, Inserm U1301, Toulouse, France; 5 Service de Biologie Cellulaire, IFB, CHUT, Toulouse, France; University of Iowa, UNITED STATES OF AMERICA

## Abstract

HIV-1-infected macrophages participate in viral transmission, dissemination, and establishment of tissue virus reservoirs. Despite counteracting viral proteins (Vif, Vpu, Vpr and Nef), cell-free virus macrophage infection is restricted by host cell factors, including those induced by interferons. Here, we show that these viral proteins and type I interferon do not influence HIV-1 cell-to-cell transfer to macrophages by cell-cell fusion with infected T cells, still leading to the formation of multinucleated giant cells (MGCs). Accordingly, depletion of SERINC5 and APOBEC3G do not alter virus spreading and formation of virus-producing MGCs. We further show that the nuclei derived from infected T cells remains transcriptionally active in MGCs and may explain resistance to restriction factors and antiretroviral drugs. Unexpectedly, we detect viral DNA in myeloid nuclei shortly after the initial fusion with macrophages. Together, these findings unravel how HIV-1 macrophage infection by cell-cell fusion escapes type I interferon and cellular restriction factors independently of the viral auxiliary proteins, while displaying resistance to antiretroviral drugs.

## Author summary

Macrophages participate in all steps of HIV-1 infection, virus transmission and dissemination, as well as in the establishment of the tissue virus-reservoirs representing major obstacles to virus eradication. The work reported here provides significant findings highlighting mechanisms by which HIV-1 evades host innate immune responses and antiretroviral therapies through viral cell-to-cell spreading in macrophages. We demonstrate that the mechanism for HIV-1 cell-to-cell infection of macrophages from infected T cells is resistant to type I interferon and host cell restrictions factors, independently of the viral accessory proteins, while displaying resistance to antiretroviral drugs. Hence, our results contribute to modify the understanding of viral persistence and immune evasion in people living with HIV.

## Introduction

Macrophages contribute to all stages of HIV-1 pathogenesis [1], including virus transmission, dissemination, and viral persistence by virus tissue reservoirs [2–7]. These virus reservoirs are a major obstacle to virus eradication in people living with HIV [4,8–13]. Infected macrophages are found in a wide range of tissues, including the central nervous system (CNS), lymph nodes, spleen, lungs, and digestive and genitourinary tracts [11]. Remarkably, infected macrophages are frequently detected as multinucleated giant cells (MGCs) [14–24]. Moreover, animal models confirmed the widespread infection of macrophages, including as MGCs, and their role in maintenance of virus reservoirs [25–28].

Paradoxically, macrophages are difficult to infect *in vitro* by cell-free viruses [29–31]. This blockage is mainly related to expression of host cell restriction factors inhibiting various steps of the viral life cycle. Acting on reverse transcription, SAMHD1 is the most studied factor restraining HIV-1 replication in macrophages [32–35], but other factors have been identified, such as SERINC proteins, and type-I interferon(IFN-I)-stimulated APOBEC3 and BST-2 proteins [32–36]. However, these cellular restriction factors are counteracted by HIV-1 Vif, Nef, Vpu and Vpr auxiliary proteins, and HIV-2 Vpx [36]. Deletion of these viral genes impacts cell-free virus replication in target cells including macrophages [37]. Nef, Vif, and Vpr, restrict host factors acting on the early steps of the virus life cycle, and only Vpu acts at a late step to promote virus release [38]. While virus replication and restriction in CD4 + T cells have been extensively studied, there is a paucity regarding viral auxiliary proteins and restriction factors in macrophage virus replication. Additionally, most of the studies on restriction factors and viral proteins utilized cell-free viruses.

Besides cell-free infection, HIV-1 cell-to-cell transfer between infected virus-donor and recipient cells represents a mode of virus dissemination for infection of lymphoid and myeloid cells [39]. Accordingly, we identified a new route for HIV-1 cell-to-cell spreading from infected CD4 + T cells to macrophages by cell-cell fusion [33,40–44]. First, infected T cells fuse with macrophages, which subsequently fuse with surrounding non-infected macrophages resulting in the formation of MGCs. *In vitro*, this rapid cell-cell fusion processes escape the SAMHD1 activity, leading to virus spread and production of high levels of infectious viruses [33,40,42,43].

In this study, we investigate the interplay between HIV-1 auxiliary proteins, restriction factors, and innate immunity in macrophage infection by cell-cell fusion. We show that deletions of Nef, Vif, Vpu, and Vpr do not influence HIV-1 transfer to macrophages. Moreover, IFN-I treatment fails to inhibit virus cell-to-cell spreading in macrophages and MGC formation. In agreement, depletion of SERINC5 and APOBEC3G does not alter virus transfer and formation of virus-producing MGCs. These findings relate to the maintenance of transcriptionally active nucleus from infected T cells in MGCs, together with the presence of viral DNA in both T cell and myeloid nuclei. These results highlight a very efficient and unconventional route of viral spread.

## Results

### HIV-1 auxiliary proteins do not influence cell-to-cell infection of macrophages by cell-cell fusion with infected T cells

**Fig 1:**
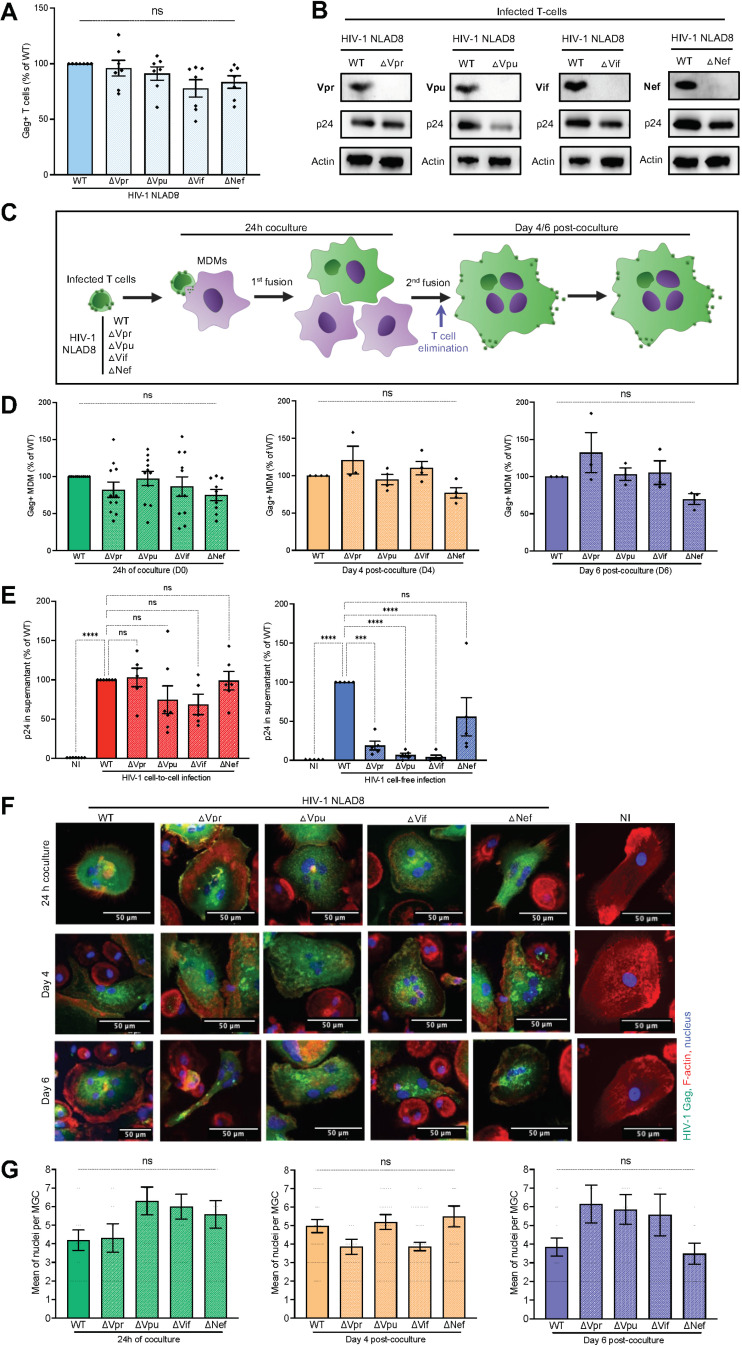
Influence of HIV-1 auxiliary proteins in virus cell-to-cell transfer from infected CD4 T cells by cell-cell fusion with macrophages. (A and B) Jurkat cells were infected with the WT HIV-1 NLAD8 or the mutated viruses deleted of each viral protein, and then analyzed 36 h later by flow cytometry after intracellular Gag staining (A) or by Western blotting (B) on cell lysates using antibodies targeting each viral protein, anti-Gag (p24) and anti-actin antibodies. In (A), the results are the means of 6 independent experiments, and are expressed as the percentages of Gag+ Jurkat relative to the percentage determined with the WT virus (100%). Each dot corresponds to an individual experiment. (C) Experimental design for analysis of HIV-1 cell-to-cell transfer from infected CD4 T cells to MDMs (created with Biorender). (D) Jurkat T cells infected with the WT or mutated viruses were cocultured for 24 h with MDMs. After T cell elimination, MDMs were analyzed immediately or cultured for 4 or 6 additional days before quantification of the percentages of Gag + MDMs by flow cytometry. Results are the means of at least 3 independent experiments performed with MDMs from at least 3 different blood donors, and are expressed as the percentages of Gag + MDMs relative to that determined by coculture of MDMs with Jurkat cells infected with WT viruses (100%). Each dot corresponds to an individual donor. (E) MDMs were infected by coculture with infected Jurkat T cells (left panel) or exposed to cell-free viruses (right panel) for 24 h. After elimination of T cells or cell-free viruses, cell-culture supernatant from MDMs were collected 6 days later, and the viral production was quantified (p24). The results were expressed as the means of p24 production quantified from MDM supernatants of at least 5 independent experiments from at least 5 different donors. NI, MDMs cocultured with noninfected Jurkat cells. Each dot corresponds to an individual donor. (F and G) Jurkat cells infected with WT or mutated NLAD8 viruses were cocultured for 24 h with MDMs. After T cell elimination, MDMs were stained immediately after coculture or 4 and 6 days later, with anti-Gag (green) antibodies, phalloidin (F-actin, red), and the nuclei with Dapi (blue), before observation by confocal microscopy. The total number of nuclei (Dapi+) per Gag + MDM was quantified on at least 50 cells. In F), representative images are shown. In G), results are expressed as the means of nucleus number per Gag + MGC. NI, MDMs cocultured with noninfected Jurkat cells. Error bars represent 1 standard error of the mean (SEM). Statistical significance was determined using the Anova test, and *P* values were obtained by Dunnett’s post-test correction (ns, *P* > 0.05; ***, P < 0.001; ****P < 0.0001).

To investigate whether Nef, Vpr, Vif and Vpu modulated viral cell-to-cell transfer from infected CD4 T cells to macrophages, mutated viruses respectively deleted of each viral protein were generated. These viruses were used to infect Jurkat cells serving as virus-donor T cells (Fig 1A and 1B). As schematized in Fig 1C, these infected Jurkat cells were then used for analyzing virus cell-to-cell transfer in MDMs by coculture for 24 h, as described previously [40,43]. After elimination of T cells, Gag + MDMs were quantified by flow cytometry just after coculture (D0), and 4 (D4) and 6 (D6) days after coculture (Fig 1D). Surprisingly, no differences in virus cell-to-cell transfer and spreading to MDMs were observed between WT and deleted viruses. Similarly, no differences were evidenced in virus production quantified in the MDM cell-culture supernatant (Fig 1E, left panel, and S1A and S1B Fig). By contrast, virus spreading and production were affected when MDMs were infected with deleted cell-free viruses (Fig 1E, right panel, and S1C and S1D Fig). The exception was the Nef-deleted viruses, inducing a slight but not significant decrease in virus production by cell-free infected MDMs compared to WT viruses (Fig 1E).

Since we previously showed that HIV-1 cell-to-cell transfer is mainly related to cell-cell fusion of infected T cells with macrophages [43], we evaluated whether cell fusion was affected when T cells were infected with deleted viruses. Infected Jurkat cells were cocultured for 24 h with MDMs before visualization of Gag+ cells by confocal microscopy, just after coculture, or 4 and 6 days after T cell elimination. All Gag + MDMs showed a diffuse Gag staining, and were multinucleated (Figs 1F, 1G and S2). These results indicate that deletion of Vif, Vpr, Nef and Vpu has no influence on HIV-1 cell-to-cell infection of macrophages by initial cell-cell fusion with infected T cells.

### HIV-1 cell-to-cell transfer in macrophages is not influenced by SERINC5

**Fig 2:**
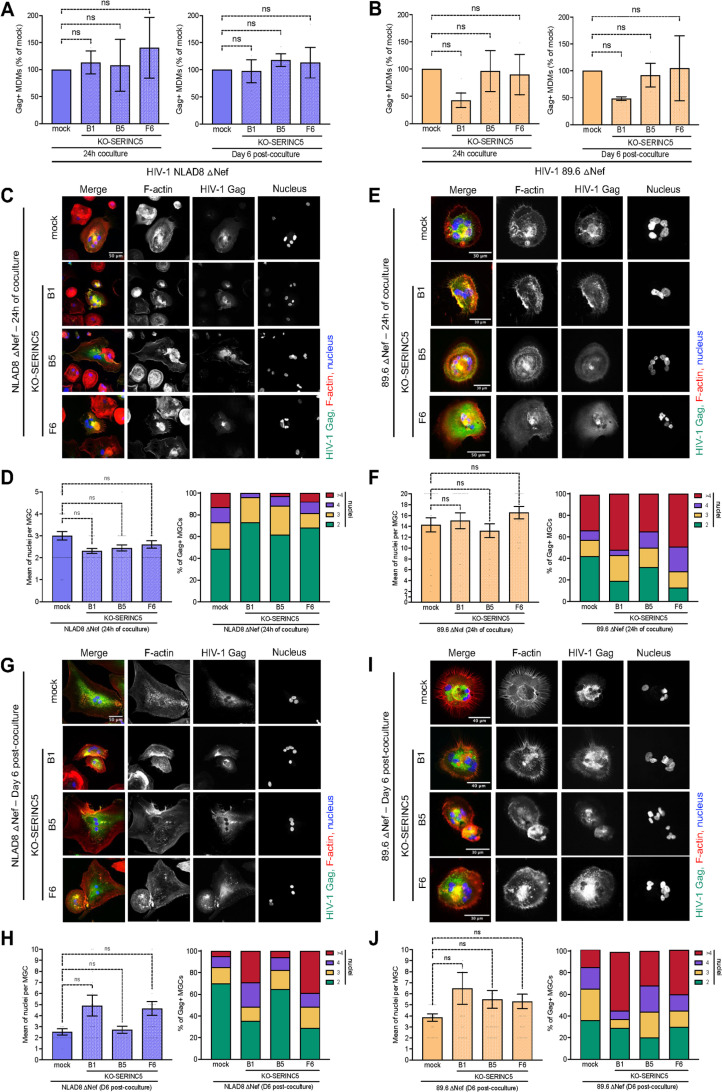
Infection of MDMs by cell-cell fusion with Serinc5-deleted infected T cells. Parental (mock) or SERINC5-KO Jurkat cells (B1, B5, and F6 clones) infected with Nef-deleted NLAD8 or 89.6 viruses were cocultured for 24 h with MDMs. After elimination of T cells, MDMs were analyzed immediately or cultured for 6 additional days. (A-B) Flow cytometry analysis. Results are the means of at least 5 independent experiments performed with MDMs from at least 5 different donors, and are expressed as the percentages of Gag + MDMs relative to that determined by coculture of MDMs with the infected parental Jurkat cells (100%). (C-J) Fluorescent confocal microscopy analysis, performed as described in Fig 1. The number of nuclei (Dapi+) per Gag + MDM was quantified on at least 50 cells. In C, E, G and I), representative images are shown. In D, F, H and J), results are expressed as the percentage of Gag + MDMs with 2, 3, 4 or more than 4 nuclei quantified from a representative experiment (right panels). In left panels), the results are expressed as the means of nuclei per Gag + MGC, and represent the means of at least 4 independent experiments performed with MDMs of 4 different donors. Error bars represent 1 SEM. Statistical significance was determined using the Anova test, and *P* values were obtained by Dunnett’s post-test correction (ns, *P* > 0.05).

A main function of Nef is to counteract SERINC proteins restricting HIV-1 entry [45,46]. To investigate the role of SERINC5, the main SERINC member restricting HIV-1, in the cell-cell fusion with infected T cells for virus cell-to-cell dissemination in macrophages, we used SERINC5-KO Jurkat clones generated via CRISPR/Cas9-assisted gene editing [47]. Since SERINC5 restriction is also dependent of the viral envelope, the 89.6 HIV-1 strain, known as very sensitive to SERINC5 [48], was included. As shown in S3 Fig, deletion of Nef in the NLAD8 or 89.6 strains had no effect on virus cell-to cell infection of macrophages by initial cell-cell fusion with infected T cells. Parental or SERINC5-KO Jurkat cells (B1, B5, and F6 clones) were then infected with WT or Nef-deleted NLAD8 and 89.6 viruses (S4A and S4B Fig), and then cocultured for 24 h with MDMs (Figs 2 and S4C–S4L). Virus cell-to-cell transfer and MGC formation were analyzed by flow cytometry and confocal microscopy after coculture, or 6 days after T cell elimination. By flow cytometry, no differences in virus cell-to-cell infection of MDMs were observed when SERINC5-KO Jurkat cells were compared to the parental cells, even when the SERINC5-KO clones were infected with the Nef-deleted 89.6 strain (Fig 2A and 2B). Accordingly, parental and KO-SERINC5 Jurkat cells infected with Nef-deleted (Fig 2C–2J) or WT (S4C and S4L Fig) viruses equally formed Gag + MGCs. These results indicate that SERINC5 suppression in virus-donor T cells does not impact cell-cell fusion for HIV-1 infection of MDMs.

### HIV-1 cell-to-cell spreading in macrophages and MGC formation are not affected by interferon-alpha and escape APOBEC3G restriction

**Fig 3:**
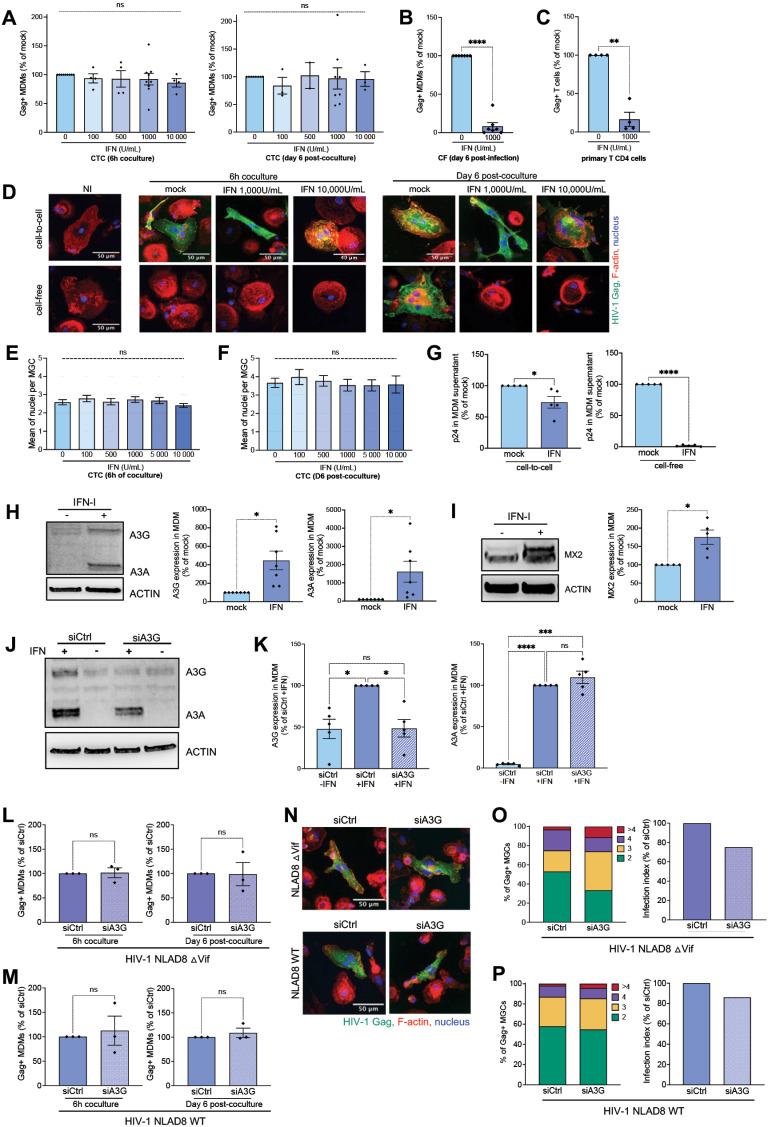
Sensitivity to type I IFN and role of APOBEC3G in HIV-1 cell-to-cell infection of macrophages by cell-cell fusion with infected T cells. (A) Infected primary CD4 T cells were cocultured for 6 h with autologous MDMs (CTC) pretreated overnight with the indicated concentration of IFNα2b. After T cell elimination, MDMs were stained for intracellular Gag and analyzed by flow cytometry just after the 6 h of coculture (left panel) or cultured for 6 additional days (right panel) with or without IFNα. Results are the means of at least 2 independent experiments performed with MDMs from at least 2 different donors, and are expressed as the percentages of Gag + MDMs relative to that determined by coculture of MDMs with infected CD4 T cells without IFNα (100%). Each dot corresponds to an individual donor. (B and C) MDMs (B) or primary CD4 T cells (C) were infected with cell-free (CF) viruses with or without IFNα (1,000 U/mL), and analyzed by flow cytometry after intracellular Gag staining 6 days later. Results are the means of at least 4 independent experiments performed with MDMs and CD4 T cells from at least 4 different donors, and are expressed as the percentages of Gag + MDMs or CD4 T cells relative to that determined by cell-free infection without IFNα (100%). (D-F) MDMs were inoculated with cell-free viruses or cocultured (CTC) with autologous infected T cells for 6 h without (mock in D) or with the indicated concentration of IFNα. After elimination of T cells or cell-free viruses, MDMs were analyzed by fluorescence microscopy just after coculture, or cultured for 6 additional days. The number of nuclei per Gag + MDM was quantified on at least 50 cells. Representative images are shown in (D). NI, MDMs were cocultured with noninfected CD4 T cells. In E and F), results are expressed as the means of nucleus number per Gag + MGC, and represent the means of at least 4 independent experiments performed with MDMs of 4 different donors. (G) MDMs were cocultured with infected CD4 T cells (left panel) or infected by cell-free viruses (right panel), for 6 h without (mock) or with IFNα (1,000 U/mL). After elimination of viruses or T cells, virus production was analyzed 6 days later from MDM supernatants from 5 independent experiments performed with MDMs of 5 different donors. Results are expressed as the percentages of p24 production relative to that determined without IFNα (mock, 100%). Each dot corresponds to an individual donor. Error bars represent 1 SEM. Statistical significance was determined using the Mann-Whitney U-test (*, *P* < 0.05; ****, *P* < 0.0001). (H and I) MDMs were cultured overnight without (mock) or with IFNα (1,000 U/mL), and then analyzed by Western blotting with antibodies against APOBEC3G and 3A (H), or MX2 (I). Representative experiments are shown in left panels. Right panels correspond to the quantification of the band intensities, and the signals measured in IFN-treated MDMs were normalized to the signal obtained for actin. Results are expressed as the percentages of the signal intensity measured with IFNα relative to the signal measured without (mock, 100%), and are means of at least 5 independent experiments performed with MDMs of at least 5 different donors. Each dot corresponds to an individual donor. Error bars represent 1 SEM. Statistical significance was determined using the Mann-Whitney U-test (*, *P* < 0.05). (J and K), Western blot analysis of lysates of siRNA-transfected MDMs (control siCtrl or siA3G) using anti-APOBEC3G and -3A, and anti-Actin antibodies. A representative experiment is shown in J). In K), results correspond to the quantification of the band intensities, and the signals measured were normalized to the signal obtained for actin. Results are expressed as the percentages of the signal intensity measured in MDMs treated or not with IFNα and transfected with siRNA relative to the signal measured in IFN-treated MDMs transfected with siCtrl (100%). Results are the means of 5 independent experiments performed with MDMs of 5 different donors. Each dot corresponds to an individual donor. Bars represent 1 SEM. Statistical significance was determined using the Mann-Whitney U-test (ns, *P* > 0.05; *, *P* < 0.05; ***, *P* < 0.001****, *P* < 0.0001). (L and M) After coculture of MDMs with ∆Vif (L) or WT-infected (M) CD4 T cells, MDMs were analyzed after coculture (6 h) or 6 days later by flow cytometry after intracellular Gag staining, and the results are expressed as the percentages of Gag+ cells related to the percentage measured in IFN-treated MDMs transfected with siCtrl (100%). Results are the means of 3 independent experiments performed with MDMs of 3 different donors. Each dot corresponds to an individual donor. Error bars represent 1 SEM. Statistical significance was determined using the Mann-Whitney U-test (ns, *P* > 0.05). (N-P) After 6 h of coculture of siRNA-transfected MDMs with ∆Vif- or WT-infected CD4 T cells, MDMs were analyzed by fluorescence microscopy, and the number of nuclei per Gag + MDM was quantified on at least 50 cells. Representative images are shown in (N). In O and P), results are expressed as the levels of MDM infection (infection index, right panels), or as the percentage of Gag + MDMs with 2, 3, 4 or more than 4 nuclei (left panels), quantified from a representative experiment of 3 independent experiments performed with MDMs of 3 different donors.

Since expression of some host restriction factors is induced by IFN-I [37,49], we next investigated the susceptibility to IFNα of HIV-1 cell-to-cell macrophage infection. MDMs were pretreated with increasing concentrations of IFNα, and then cocultured with primary autologous infected CD4 + T cells as previously. MDMs were then analyzed by flow cytometry and confocal microscopy, just after coculture or 6 days later (Fig 3). Surprisingly, macrophage infection by virus cell-to-cell transfer was not affected by IFNα, even at the highest concentration (10,000 Unit/mL) (Fig 3A). In contrast, cell-free MDM infection was strongly inhibited by 1,000 Unit/mL of IFNα or lower concentration (Figs 3B, S5A and S5E). Cell-free infection of primary CD4 T cells was also affected by IFNα (Fig 3C). By contrast, cell-cell fusion between infected T cells and MDMs, as well as MGC formation were not affected by IFN-I (Figs 3D–3F and S5I–S5M) compared to cell-free virus infection (S5B–S5D and S5F–S5M Fig). Accordingly, virus production assessed 6 days after coculture was moderately affected by treatment with 1,000 Unit/mL of IFNα (Fig 3G, left panel), whereas cell-free virus MDM infection was totally inhibited (Fig 3G, right panel). These findings demonstrate that HIV-1 cell-to-cell spreading in macrophages through cell-cell fusion is not sensitive to IFN-I. Since HIV-1 restriction is largely governed by IFN-I-stimulated cellular factors such as APOBEC3 cytidine deaminases [49], we confirmed the stimulation of APOBEC3G and 3A expression in MDMs treated with 1,000 Unit/mL of IFNα (Fig 3H). Similarly, expression of the IFN-stimulated Mx2 protein, a factor inhibiting viral DNA nuclear translocation [50], was also upregulated in IFNα-treated MDMs (Fig 3I).

Since APOBEC3G is a potent restriction factor for HIV-1 replication in primary cells [49,51,52], we evaluated APOBEC3G restriction in HIV-1 cell-to-cell spreading from infected primary CD4 T cells to IFNα-stimulated macrophages. Differentiated MDMs were transfected with siRNA targeting APOBEC3G (siA3G), before pretreatment with 1,000 Unit/ml of IFNα, and then cocultured with infected T cells as schematized on S6A Fig. As evidenced in Fig 3J and 3K, the IFNα-induced APOBEC3G upregulation was reduced by siA3G, leading to expression equivalent to the unstimulated MDMs transfected with siCtrl (Fig 3J and 3K, left panel). By contrast, IFNα-induced APOBEC3A upregulation was not affected by siA3G (Fig 3J and 3K, right panel). APOBEC3G-depleted MDMs were then pretreated with IFNα, and cocultured with primary T cells infected with ΔVif or WT viruses. No differences in virus cell-to-cell infection of IFNα-stimulated MDMs were observed in APOBEC3G-depleted MDMs analyzed by flow cytometry, immediately after coculture or 6 days later (Fig 3L and 3M). Similarly, no differences in MGC formation were observed with IFNα-stimulated MDMs transfected with siA3G or siCtrl, either cocultured with WT or ΔVif infected T cells (Figs 3N–3P, S6B and S6C). These results show that cell-to-cell infection of IFNα-stimulated macrophages by formation of MGCs is not restricted by APOBEC3G. This finding also suggests that other IFN-stimulated APOBEC3 proteins do not affect cell-to-cell infection of macrophages by cell-cell fusion.

### The T cell-derived nucleus of MGCs is transcriptionally active

**Fig 4:**
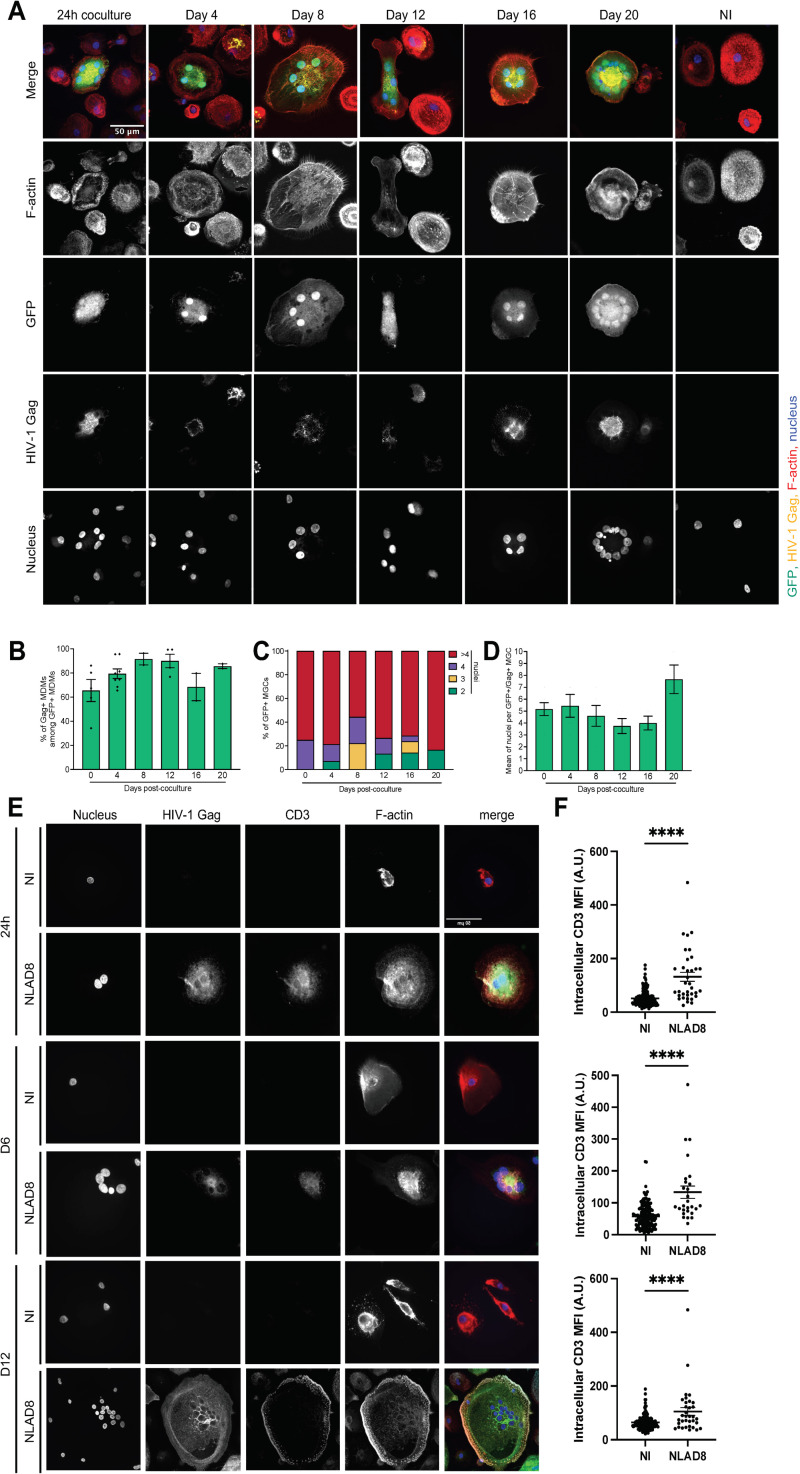
Maintenance of transcriptionally-active T cell nuclei in MGCs formed upon cell-cell fusion. Jurkat-LTR-GFP (A-D) or purified CD4 + T (E and F) cells were infected with HIV-1 NLAD8, and then cocultured with MDMs. After elimination of T cells, MDMs were analyzed immediately (24 h, or day 0) or cultured for different period of time (until day 12 or 20) before analysis by fluorescence microscopy after staining with anti-Gag (yellow brown) antibodies, phalloidin (F-actin, red), GFP or Gag (green), and Dapi (Nucleus, blue). The total number of nuclei (Dapi+) per Gag + MDM was quantified on at least 50 cells. (A and E) Representative images are shown. In B) Results correspond to the percentage of Gag+ cells among GFP + MDMs. Each dot corresponds to an individual donor. In C) Results are expressed as the percentage of Gag + /GFP + MDMs with 2, 3, 4 or more than 4 nuclei quantified from a representative experiment. In D) Results are expressed as the means of the nucleus number per Gag + /GFP + MDM, and represent the means of at least 4 independent experiments performed with MDMs of 4 different donors. Error bars represent 1 SEM. (E and F) Fluorescence microscopy analysis of the results obtained from a representative experiment performed on MDMs, just after 24 h coculture with primary CD4 T cells, or 6 or 12 days after the initial coculture and elimination of T cells. Cells were then stained with anti-CD3, anti-Gag, and DAPI (nuclei) and analyzed by confocal microscopy. Representative images are shown in E). In F), intracellular CD3 mean fluorescence intensities (MFI) were quantified as indicated in Materials and Methods. Each dot corresponds to 1 cell, and at least 30 cells were analyzed for each condition. Horizontal bars represent means + /- 1 SEM. Statistical significance was determined with the Mann-Whitney U-test (****, *P* < 0.0001).

To characterize the mechanisms responsible for bypassing the cellular restriction factors independently of the viral auxiliary proteins, as well as the resistance of MGC formation to IFN-I, we investigated the maintenance of the T-cell nucleus containing proviral DNA. Therefore, we first used the Jurkat-LTR-GFP cell-line, expressing GFP under the control of the viral promoter, as virus-donor T cells. About 80% of infected Gag+ Jurkat-LTR-GFP cells were co-expressing GFP (S7A Fig). We then cocultured infected Jurkat-LTR-GFP cells with MDMs for 24 h and the MGC formed expressing Gag were monitored for GFP expression (S7B Fig). As shown in Fig 4A, Gag + MGCs expressing GFP could be detected even 20 days after the initial coculture of infected T cells with MDMs. A large majority of the GFP+ cells were Gag+ (75–95%, Fig 4B) and contained several nuclei (Fig 4C and 4D). Representative experiments of the proportion of GFP + , Gag + and double Gag + /GFP+ cells quantified 12 and 20 days after coculture, are shown in S7C–S7F and S7G–S7J Fig, respectively. 12 days post-coculture, around 50% of the Gag + MGCs were GFP+ (S7E Fig), and these double Gag + /GFP+ cells were multinucleated (S7E and S7F Fig, right bars). Around 35% of the Gag + MGCs were GFP- (S7F Fig), and could correspond to MDMs infected with cell-free viruses released by the Gag + MGCs during the 12 days of culture [40]. In agreement, around 40% of the Gag + /GFP- cells were mononucleated (S7E Fig). Similar results were obtained on infected MGCs co-expressing Gag and GFP 20 days after the initial coculture (S7G–S7J Fig). Since GFP was expressed only from the integrated LTR-GFP of T cell nuclei, these results show that these nuclei remain transcriptionally active in most MGCs.

To further confirm the transcriptional activity of the T cell nuclei in MGCs, we used purified primary CD4 T cells as virus-donor T cells. As previously, infected primary T cells were cocultured with MDMs for 24 h, and expression of the specific CD3 T cell marker was followed after T cell elimination, just after the coculture, and 6 and 12 days later. As shown in Fig 4E and 4F, the CD3 T cell specific marker was initially detected just after coculture, but also 12 days later, confirming that the T cell nuclei remain transcriptionally active in most MGCs.

### HIV-1 cell-to-cell spread in macrophages is resistant to antiretroviral drugs

**Fig 5:**
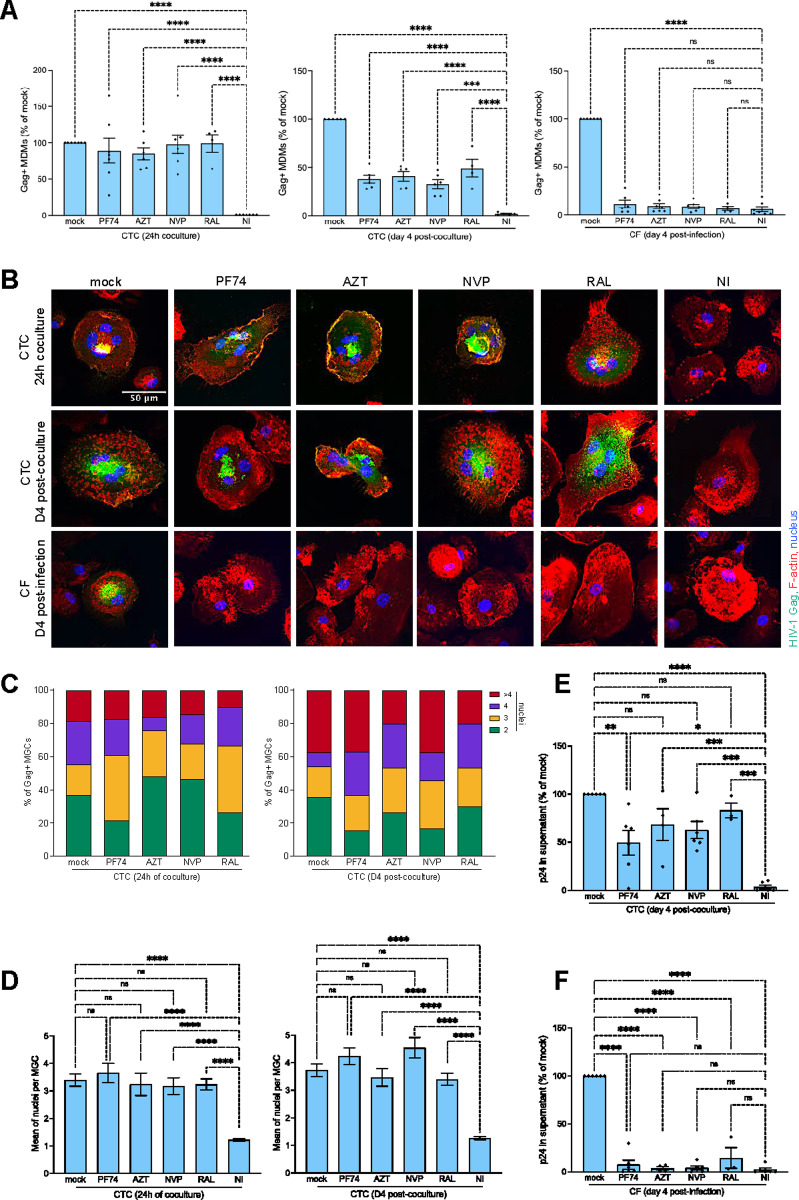
Sensitivity to antiretroviral drugs of HIV-1 cell-free and cell-to-cell infection of macrophages. (A) MDMs pretreated with AZT, NVP, RAL or PF74 were infected with cell-free viruses (right panel) or cocultured for 24 h with infected Jurkat cells and analyzed just after coculture (left panel) or 4 days later (middle panel) by flow cytometry after intracellular Gag staining. Results are the means of at least 5 independent experiments performed with MDMs from at least 5 different donors, and are expressed as the percentages of Gag + MDMs relative to that determined without drugs (mock, 100%). Each dot corresponds to an individual donor. Error bars represent 1 SEM. Statistical significance was determined using the Anova test, and *P* values were obtained by Dunnett’s post-test correction (ns, *P* > 0.05; *, *P* < 0.05; ***, *P* < 0.001; ****, *P* < 0.0001). (B-D) MDMs pretreated with PF74, AZT, NVP, and RAL or without (mock) were inoculated with cell-free viruses (CF) or cocultured (CTC) with infected Jurkat cells for 24 h. After T cell elimination, MDMs were analyzed by fluorescence microscopy just after the coculture, or cultured for 4 additional days with or without antiretroviral drugs. The total number of nuclei per Gag + MDM was quantified on at least 100 cells. Representative images are shown in (B). NI, MDMs cocultured for 6 h with noninfected CD4 T cells. In C), results are expressed as the percentage of Gag + MDMs with 2, 3, 4 or more than 4 nuclei quantified from a representative experiment. In D), results are expressed as the means of nucleus number per Gag + MGC, and represent the means of at least 4 independent experiments performed with MDMs of 4 different donors. (E and F) MDMs pretreated or not with antiretroviral drugs were cocultured with infected CD4 T cells (E, CTC) for 24 h or infected by cell-free viruses (F, CF). After elimination of the virus inoculum or T cells, virus production was analyzed 4 days later from MDM supernatants of 6 independent experiments performed with MDMs of 6 different donors. Results are expressed as the percentages of p24 production relative to that determined without drugs (mock, 100%). Each dot corresponds to an individual donor. Error bars represent 1 SEM. Statistical significance was determined using the Anova test, and *P* values were obtained by Dunnett’s post-test correction (ns, *P* > 0.05; **, *P* < 0.01; ***, P < 0.001; ****, *P* < 0.0001).

Since the T cell nucleus remains transcriptionally active in MGCs, we investigated whether this mode of infection might also impact on the susceptibility of antiretroviral drugs targeting early steps of virus replication. MDMs were pretreated with antiretroviral drugs before coculture with infected Jurkat cells, including zidovudine (AZT) and nevirapine (NVP) as nucleoside and non-nucleoside reverse-transcriptase inhibitors (NRTI and NNRTI, respectively), raltegravir (RAL) as integrase inhibitor (INSTI), and PF74 as a capsid-targeting compound [53,54]. After coculture, MDM infection was first analyzed by flow cytometry. As shown in Fig 5A (left panel), there was no effect of antiviral drugs on virus transfer when Gag + MDMs were analyzed just after coculture. Interestingly, only a partial decrease of Gag + MDMs, statistically significant compared to non-infected cells (NI), was observed when infection was analyzed 4 days later (central panel). By contrast, a total inhibition was observed when MDMs were infected by cell-free viruses (right panel). Similarly, when analyzed by fluorescence microscopy (Figs 5B–5D, S8A and S8B), MGCs were formed just after the coculture and 4 days later even in the presence of antiretroviral drugs. By contrast, a complete inhibition of the low level of MGCs formed was observed when MDMs pretreated with antiviral drugs were infected by cell-free viruses, and almost all the MDMs were mononucleated (Figs 5B and S8C–S8E). Accordingly, viral production was partially resistant to antiviral drugs when MDMs were infected by virus cell-to-cell transfer (Fig 5E), whereas it was abolished by cell-free infection (Fig 5F). These results confirm that bypassing the early steps of the viral life cycle through transfer of viral material and infected T cell nuclei, result in a significant resistance to antiretroviral drugs.

### Both lymphoid and myeloid nuclei of MGCs contain HIV-1 DNA

**Fig 6:**
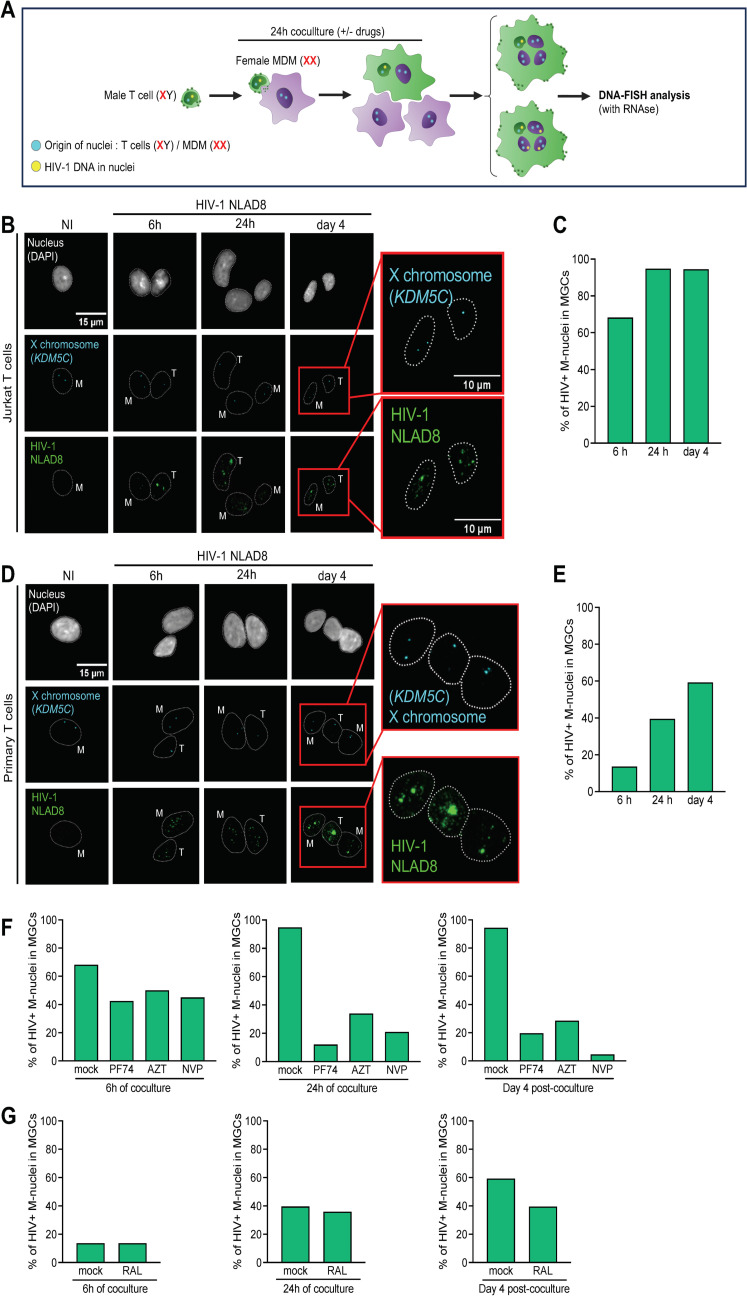
FISH analysis of HIV-1 DNA in lymphoid and myeloid nuclei of MGCs. (A) Schematic representation of the experimental design for detection of viral DNA in MGC nuclei (created with Biorender). (B-E) MDMs were cocultured for 6 or 24 h with non-infected- or NLAD8-infected Jurkat (in panels B and C) or primary T (in panels D and E) cells, or cultured after elimination of T cells for 4 additional days before DNA-FISH analysis. MDMs were fixed, permeabilized, stained with Dapi, and then incubated after RNAse treatment with specific probes for detection of the *KDM5C* gene located on the X chromosome together with the HIV-NLAD8 probe for detection of the proviral DNA. Representative images of non-infected mononucleated (left images, NI) and infected multinucleated MDMs (6 or 24 h after coculture, or cultured for 4 additional days), are shown. Enlargements from the right images marked with a red rectangle are shown. The outlines of nuclei are indicated by dotted lines; T and M correspond to nuclei of T cell and myeloid origins containing a single and two X chromosomes, respectively. In C and E), quantification of the percentage of MGCs with MDM nuclei containing HIV-positive dots after 6 or 24 h of coculture, or 4 days later. The number of HIV-positive M-nuclei was quantified in at least 30 MGCs. (F and G) MDMs pretreated or not with antiretroviral drugs (PF74, AZT, NVP or RAL) were cocultured for 6 or 24 h with NLAD8-infected Jurkat (panel F) or primary CD4 T (panel G) cells, or cultured for 4 additional days in the presence of the drugs before DNA-FISH analysis. After elimination of T cells, MDMs were fixed, permeabilized, stained with Dapi, and then incubated with probes for detection of the *KDM5C* gene together with the HIV-NLAD8 probe. Representative images of infected multinucleated MDMs after 6 or 24 h of coculture, or 4 days later are shown in S9B and S9C Fig. Results are expressed as the percentage of MGCs HIV + M-nuclei containing HIV-positive dots after 6 or 24 h of coculture, or 4 days later, in non-treated (mock) or treated MGCs. The number of HIV-positive M-nuclei was quantified in at least 30 MGCs.

Since some T cell nuclei remain transcriptionally active in the Gag-positive MGCs, we questioned whether copies of viral DNA were present only in T cell nuclei or might be also found in myeloid nuclei. DNA-FISH experiments were performed to visualize viral DNA in MGC nuclei. As schematized in Fig 6A, Jurkat cells derived from a male with a T cell leukemia [55], or primary CD4 T cells purified from a male blood donor, were used as virus-donor T cells as previously, while MDMs differentiated from female donor monocytes were used as macrophage targets. To distinguish between lymphoid and macrophage nuclei, a probe targeting the *KDMC5* X chromosome gene was employed [56]. Jurkat or primary T cells were infected and then cocultured for 6 or 24 h with MDMs, and DNA-FISH was performed just after cocultures or 4 days later. As expected, when female MDMs were cocultured with non-infected Jurkat cells, mononucleated non-infected MDMs exhibited 2 copies of the *KDMC5* gene (Fig 6B and 6D, NI). By contrast, MGCs formed upon coculture with infected Jurkat cells or male primary T cells containing a single X chromosome (T) could be discriminated from the myeloid nuclei (M) (Fig 6B and 6D). When MDMs were stained with viral DNA probes generated from the full length proviral NLAD8 strain, almost all MGC T cell nuclei contained, as expected, fluorescent dots of HIV-1 DNA as soon as 6 h of coculture (S9A Fig). Interestingly, viral DNA dots were also detected in some myeloid nuclei as soon as 6 h of coculture (Fig 6B–6E), suggesting that viral DNA present in the cytoplasm of infected T cell can be translocated into the macrophage nuclei. Then, the number of myeloid nuclei containing viral DNA significantly increased after 24 h of coculture or 4 days later (Fig 6C and 6E). Pre-treatment of MDMs with antiretroviral drugs such PF74, AZT, NVP and RAL, resulted in a slight (at 6 h) or a significant (at 24 h of coculture and 4 days later) decrease of myeloid nuclei containing viral DNA (Fig 6F and 6G), showing the specificity of the viral DNA detection. These observations and findings show that both T cell and myeloid nuclei of MGCs can contain viral DNA copies, and can explain the high virus-production of MGCs.

## Discussion

Here, we demonstrate that the main mechanism promoting HIV-1 cell-to-cell infection of macrophages [33,40,42,43] is independent of the viral Vpr, Vif, Vpu and Nef proteins. Additionally, antiviral IFN-I has no effect on virus cell-to-cell infection of macrophages after cell-cell fusion with infected T cells. In agreement, formation of virus-productive MGCs escapes the viral restriction induced by the IFN-stimulated APOBEC3 deaminases [49]. Finally, by showing that the T cell nuclei remain transcriptionally active in MGCs, together with the presence of viral DNA in both T cell and myeloid nuclei of early formed MGCs, we highlight how cell-cell fusion is so efficient for virus spreading in macrophages [33,40–43], and resistant to antiretroviral drugs. It is difficult to infer what happens *in vivo* in lymphoid and non-lymphoid tissues of people living with HIV under effective cART, since there is no study investigating in real time tissues of infected patients. Investigation of the formation of MGCs in tissues of experimentally infected monkeys could help to understand the mechanisms of MGC formation in different tissues in monkeys treated or not with efficient cART. To our knowledge, only 2 studies from the same group reported that myeloid cells from spleen and lymph nodes of SIV-infected macaques contain T cell markers and viral RNA and DNA originating from infected T cells, agreeing with the model we propose in the present study [57,58].

HIV-1 Vif, Nef, Vpr, Vpu, and HIV-2 Vpx, hijack a plethora of cellular functions [59], but they mainly contribute in counteraction of cellular restriction factors. It is well established that deletion of these viral genes impacts cell-free virus replication in target cells, including macrophages [36]. However, Nef is a notable exception and seems dispensable for virus replication in macrophages in both cell-free and cell-to-cell infection. While deletion of Nef failed to affect macrophage infection, it was reported that it profoundly disturbed macrophage functions, such as migration and phagocytosis [60–62].

We previously showed that SAMHD1 did not restrict HIV-1 cell-to-cell spreading from infected T cells to macrophages in the absence of HIV-2 Vpx [33]. Interestingly, we now extend that deletion of HIV-1 Nef, Vif, Vpr and Vpu, counteracting SERINC, APOBEC3, and BST-2, have no impact on cell-to-cell infection of macrophages by fusion with live infected T cells. These findings indicate that macrophage infection by cell-cell fusion bypasses restriction factors independently of the viral auxiliary proteins. A single study regarding the role of Vpu on virus cell-to-cell macrophage infection reported that it could enhance the susceptibility of macrophages to infection [63], but this effect was related to the phagocytosis of dying infected T cells [64].

SERINC proteins impair virus infectivity through modulation of cell-free virus entry in T cells [45,65–67], but almost nothing was reported regarding these cellular proteins for HIV-1 restriction in macrophages. SERINC proteins have been shown as factors able to decrease the fusogenicity of certain viral envelope by acting as a phosphatidylserine flippase [46]. In agreement with the lack of Nef requirement in virus cell-to-cell transfer, we now report that SERINC5 deletion in infected T cells, has no impact on fusion of ∆Nef-virus-infected T cells with macrophages, MGC formation and *de novo* virus production. This lack of restriction is observed even when using the highly-sensitive SERINC5 89.6 HIV-1 strain [48]. A single study using cell-free infection of MDMs with ∆Nef-viruses produced with SERINC5 showed that restriction was dependent of the monocyte donor [68].

While some restriction factors are not stimulated by IFN-I [37], most other factors such as APOBEC3 family members and BST-2 participate in HIV-1 restriction as IFN-stimulated genes (ISGs) [38,49]. In contrast to cell-free infection of macrophages and T cells, no inhibition of cell-to-cell virus transfer from infected T cells and MGC formation was observed at high IFNα concentration, demonstrating that cell-to-cell virus infection of macrophages by cell-cell fusion is resistant to IFNα. This IFN-I resistance of the cell-cell fusion infection may result from inhibition of IFNα-mediated signaling pathways [69]. The rapid transfer and mixing of membrane, cytoplasmic and nuclear contents of infected T cells [33,40] may certainly impact various steps of IFN-I pathways and ISG induction. Alternatively, since we show that APOBEC3 and Mx2 IFN-stimulated factors are induced by IFNα, but fail to limit virus spreading, our findings suggest that the cell-cell fusion mode of macrophage infection bypasses and/or overcomes the IFN-I pathways. Interestingly, other authors showed that cell-cell fusion of macrophages with infected T cells resulted in intercellular transfer of mediators of the innate immunity such as the cyclic GMP-AMP synthase leading to IFN-I production [70]. Nevertheless, these authors concurred with our findings that IFN and ISG induction upon fusion with infected T cells are not sufficient for HIV-1 inhibition in macrophages. Here, we show that IFNα is largely inactive on cell-to-cell infection and virus spreading through formation of MGCs 6 days after the initial coculture. In contrast, macrophage infection and virus production by cell-free viruses is totally inhibited by IFNα treatment. These observations suggest that only the cell-cell fusion mechanism of infection leading to MGC formation is resistant to the antiviral activity of IFNα. However, IFNα is likely still effective to inhibit infection of the remaining macrophages by the cell-free infectious viruses released by MGCs at late stage of infection, several days after the initial coculture of the macrophage targets with infected T cells.

In agreement with IFN-I resistance of cell-to-cell infection of macrophages, IFNα-induction of APOBEC3G and 3A, Mx2, and likely other IFN-induced factors such as the Vpu-counteracted Bst-2/tetherin protein, does not affect macrophage infection by cell-cell fusion. Depletion of IFN-stimulated APOBEC3G has no impact on HIV-1 transfer from infected T cells, confirming that cell-cell fusion mode of macrophage infection escapes APOBEC3 family members. By contrast, using the promonocytic THP-1 cell-line, previous studies showed that deletion of all APOBEC3 members alleviates restriction of cell-free HIV-1 infection [51,52,71]. Although we do not formally show that depletion of other APOBEC3 proteins might impact virus cell-to-cell spreading in macrophages, our findings on APOBEC3G and the lack of Vif impact and IFN-I resistance, indicate that macrophage infection by cell-cell fusion escapes restriction by all IFN-stimulated APOBEC3 family members.

**Fig 7:**
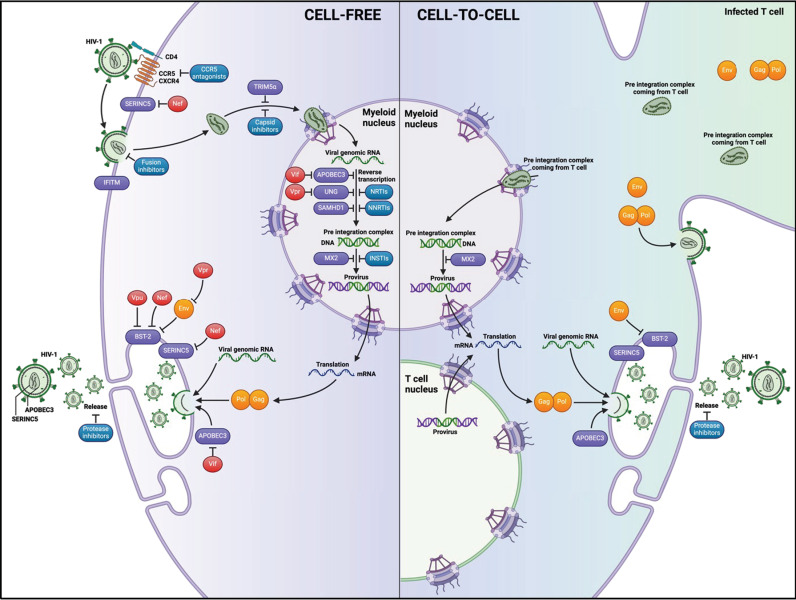
Model for HIV-1 cell-to-cell infection of macrophages through cell-cell fusion with infected T cells (created with Biorender). (Left part) Cell-free virus infection. The various early steps of the virus life cycle (before viral DNA integration), including intracellular routing, reverse-transcription of the viral RNA, nuclear translocation and integration of the viral DNA into the host chromosomes is restricted by several host cell restriction factors (light blue), often counteracted by viral auxiliary proteins (Nef, Vpr and Vif, in red). These early steps of the virus life cycle are also the main targets of cART (dark blue), including capsid inhibitors, NRTIs and NNRTIs, and INSTIs, for efficient treatment of people living with HIV. After integration, transcription of the viral genes and synthesis of genomic RNA result in *de novo* synthesis and assembly of the viral components, and then budding of the viral particles. In infected macrophages, the assembly of the viral components and then the budding of *de novo* formed virus particles specifically take place in a specialized intracellular membrane compartment called the VCC, for virus-containing compartment. The BST-2/tetherin protein, counteracted by the viral Vpu protein (in red) is the best-characterized restriction factor acting in the late steps of the viral life cycle by tethering virus particles at the cell surface of macrophages, leading to a limitation of virus budding and release. **(Right part) Cell-to-cell virus infection.** Cell-cell fusion of infected T cells with macrophages results in the rapid and massive transfer of all the T cell contents, including membrane mixing, cytoplasm content sharing, and finally transfer and maintenance of the T cell nuclei already containing integrated proviral DNA. This cell-cell fusion process allows to bypass and/or overcome the intracellular host cell restriction counteracted by the viral auxiliary proteins, as well as antiretroviral drugs acting in the early steps of the viral life cycle, leading to early virus production by the lymphocyte/macrophage fused cells formed. In addition, after cell-cell fusion and mixing of the T cell content in the newly forms fused cells, the viral reverse-transcription and/or preintegration complexes, already initiated in infected T cells, will mature and be translocated into the myeloid nuclei, and may explain how both T cell and myeloid nuclei contain viral DNA early after the cell-cell fusion process.

Our present results, as well as previous findings on SAMHD1 [33], indicate that macrophage cell-to-cell infection by cell-cell fusion with infected T cells escapes restriction by IFN-stimulated or IFN-independent restriction factors. These findings support a mechanism by which the transfer and maintenance of the T-cell nuclei containing integrated proviral DNA allow to bypass early steps of the virus replication. This leads to early viral expression and explain resistance to antiretroviral drugs of the newly formed fused cells. Our findings suggest an unsuspected mode of HIV-1 macrophage infection. Such original process allows to escape restriction by SERINC5 and APOBEC3G, and SAMHD1 [33], involved in the early steps of the viral life cycle, independently of the viral auxiliary proteins (model on Fig 7). We could speculate that proviral DNA present in the cytoplasmic fraction of infected T cells is transferred by cytoplasmic mixing for translocation in myeloid nuclei escaping restriction by host factors. This model supports high and rapid production of viruses a few hours after cell-cell fusion, as shown here and previously [33,40]. Additionally, we show that this mode of cell-to-cell macrophage infection is less susceptible to antiviral drugs targeting early steps of the viral life cycle, such as inhibitors of reverse transcription, uncoating and integration.

Interestingly, we have detected the presence of viral DNA into some macrophage nuclei, very early after the cell-cell fusion with infected T cells. Since viral DNA was detected in some myeloid nuclei even when the reverse transcription, nuclear translocation and integration steps were inhibited, we can hypothesize that it likely results from the transfer of viral reverse-transcription and/or preintegration complexes initiated in infected T cells, and subsequently translocated into the myeloid nuclei upon cell-cell fusion (see model on Fig 7). In addition, we cannot exclude that the presence of viral DNA in some M-nuclei is also partly related to macrophages infected with cell-free viruses released by the Gag + MGCs. Finally, these observations should be replaced within the new concept of late capsid uncoating, which was especially evidenced in infected macrophages [72–76].

Together with the transfer of T cell nuclei in the lymphocyte/macrophage fused cells, the translocation to myeloid nuclei of viral DNA initiated in infected virus-donor T cells may explain how HIV-1 macrophage infection by cell-cell fusion escapes restriction by cellular restriction factors [33]. This happens independently of the viral auxiliary proteins in an IFN-I-independent manner. Additionally, the maintenance of active T cell nuclei in MGCs, and the nuclear translocation of viral DNA after the cell-cell fusion, may also explain how infected MGCs are resistant to some antiretroviral drugs for high production of infectious virus particles [40]. Therefore, characterizing the mechanisms of viral spread in myeloid cells could lead to a better understanding of: i) sexual transmission of cell-associated HIV-1, ii) HIV-1 spreading between virus target cells in tissues, iii) susceptibility to antiretroviral drugs, and iv) establishment of viral macrophage tissue reservoirs [1,4,5,11]. These mechanisms are critical since shared by many other viruses, including HIV-1 and SARS-CoV-2, triggering cell-cell fusion between infected and non-infected cells, leading to a phenomenon of virus cell-to-cell spreading observed in tissues of infected patients [77,78].

## Materials and methods

### Ethics statement

Blood samples from anonymous healthy donors were purchased at “*Etablissement Français du Sang*” (EFS) (French National Blood Agency). Donors provided written informed consent to EFS at the time of blood collection (agreement #18/EFS/030). Samples were used in accordance with legal and ethical conditions we previously detailed in our previous work [42].

### Plasmids and reagents

The proviral pNLAD8 and p89.6 plasmids were obtained from the AIDS Research and Reference Reagent Program, NIAID. The Nef-deleted p89.6 ∆Nef was obtained from Bernard Lagane (Inserm, Toulouse, France), and the pVSVg plasmid encoding the VSV-G envelope glycoprotein has been described [42]. The pNLAD8 plasmids deleted from each of the HIV-1 auxiliary proteins (∆Nef, ∆Vif, ∆Vpr, and ∆Vpx) were constructed from the wild-type pNLAD8 plasmid. Briefly, pNLAD8∆Vif was constructed through insertion of the AgeI-PfIMI fragment of the pNL4.3Vif-Vpr- plasmid (obtained from the AIDS Research and Reference Reagent Program) in the pNLAD8 previously digested by AgeI and PflMI. The pNLAD8∆Vpr was constructed through insertion of the PflMI-EcoRI fragment of the pNL4.3Vif-Vpr- plasmid in the pNLAD8 previously digested by PfIMI and EcoRI. The pNLAD8∆Vpu was constructed through insertion of the EcoRI-BamHI fragment of the pNL4.3Vpu- plasmid (obtained from the AIDS Research and Reference Reagent Program) in the pNLAD8 previously digested by EcoRI and BamHI. The pNLAD8∆Nef was construct by digestion of the pNLAD8 with XhoI, filled with Klenow before religation. The SERINC5-KO Jurkat cell lines (B1, B5, and F6 clones) were obtained from Christine Goffinet (Liverpool School of Tropical Medicine, United Kingdom), and have been described and characterized previously [47]. The following antibodies were used: RD1- or fluorescein isothiocyanate (FITC)-conjugated anti-Gag (clone KC57, Beckman Coulter); Alexa Fluor 647- or Fluor 555-conjugated phalloidin (Life Technologies); while anti-Vpr, -Vif, -Nef and -Vpu, as well as anti-APOBEC3G/A (anti-Apo17) antibodies were obtained from the AIDS Research and Reference Reagent Program; anti-Mx2 was purchased from Novus Bio; and anti-b-actin was purchased from Invitrogen [33]. The antiretroviral AZT, NVP, RAL and PU74 compounds were obtained from the AIDS Research and Reference Reagent Program, while IFNα2a was purchased from Miltenyi Biotech.

### Cell culture

HEK293T and Jurkat cell lines were obtained from the ATCC, and were maintained in culture as described [42]. Monocytes were purified from blood of healthy donors using a CD14-positive selection kit (CD14 microbeads; Miltenyi) according to manufacturer’s guidelines. Monocytes were then differentiated into macrophages for 8 days in culture medium supplemented with 25 ng/ml of macrophage colony-stimulating factor (M-CSF) (Miltenyi) as described [43]. Human primary CD4 + T cells were isolated and maintained in culture as described [43].

### Virus production and titration

Wild-type or deleted HIV-1 NLAD8 and 89.6 strains were produced in HEK293T cells as previously described [42]. The amounts of viral p24 produced were determined by enzyme-linked immunosorbent assay (ELISA; Innogenetics) as recommended by the manufacturer. Viral titers were determined using Jurkat cells and flow cytometry as described previously [36]. For production of viruses dedicated to the cell-free infection experiments, viral stocks were used at a multiplicity of infection (MOI) of 0.5 to infect Jurkat cells for 16 h. After washing, cells were cultured for another 24 h, and the amount of Gag p24 produced in the cell-culture supernatant was determined by ELISA as described [42].

### Cell-free and cell-to-cell viral transfer and dissemination in MDMs

For cell-free infection of MDMs or primary CD4 T cells, viruses produced in infected Jurkat T cells were used at a concentration of 250 ng/mL of viral p24 for 10^6^ MDMs as described [43]. After 24 h of infection and elimination of the viral inoculum, the level of Gag + MDMs was analyzed by flow cytometry as previously described [42], just after the 24 h of infection or after culture for 4 or 6 additional days. For cell-to-cell virus infection assays of MDMs from infected Jurkat or primary purified CD4 T cell, cells were carried out as described previously [33,40]. Briefly, Jurkat and primary CD4 T cells were infected with virus stocks produced from HEK 293T cells at a MOI of 0.5 for 16 h, and then for another 24 h after an intermediate washing step. Where indicated, MDMs were pretreated before coculture for 1 h with antiretroviral drugs (AZT, NVP, RAL, and PF74) at the concentration of 10 ∆M, or overnight with IFNα2b at the indicated concentration (ranging from 100 to 10,000 U/mL), and then cocultured at a 2:1 cell ratio, without or with either antiretroviral drugs or IFN2αb, with infected T cells for 6 or 24h as described [42]. T cells were eliminated by washes in PBS and 4 mM EDTA-containing PBS [40]. The percentages of Gag + MDMs were analyzed by flow cytometry using the KC57 anti-Gag mAb (1/500) just after the coculture or after 4 or 6 additional days in culture [40]. Cell-culture supernatants were also harvested for quantification of the virus production by p24 ELISA as described [33,40]. Similarly, MDMs depleted of APOBEC3G after transfection of specific siRNA (Dharmacon) as described [33] were cocultured with primary CD4 T cells, and then analyzed by flow cytometry or for viral production in the cell-culture supernatant as described above.

### Fluorescence microscopy

To visualize virus cell-to-cell transfer by cell-cell fusion, infected Jurkat or primary CD4 T cells were cocultured for 6 or 24 h with MDMs plated onto coverslips as described [40,42]. Where indicated, MDMs were pretreated before coculture with IFNα2b (ranging from 100 to 10,000 U/mL) or antiretroviral drugs (AZT, NVP, RAL, or PF74 at the concentration of 10 ∆M) as described above. After elimination of T cells, MDMs were directly analyzed after the coculture or cultured for different period of time, before fixation, blockage and staining with Dapi as described [42]. MDMs were then permeabilized and stained using KC57 FITC-conjugated anti-Gag antibody (1/200 dilution), and phalloidin-Alexa Fluor 647 (Molecular Probes), diluted in permeabilization buffer for 1 h. Coverslips were then washed with PBS and mounted on slides using 10 μl of Fluoromount (Sigma). Images were acquired on a spinning disk (CSU-X1M1; Yokogawa)-equipped inverted microscope (DMI6000; Leica) and were then processed using Fiji software (ImageJ; NIH). Quantitative image analysis and determination of Dapi+ nuclei were analyzed from images of at least 100 cells followed by processing using Fiji as described [33,40]. Quantitative CD3 expression was analyzed form images on at least 50 cells after staining with FITC-conjugated KC57 anti-Gag, phalloidin-Alexa Fluor 647 and PE-conjugated anti-human CD3 (Biolegend) using Fiji software by defining a region of interest using the F-actin staining and measuring the whole fluorescence intensity of the CD3 marker in Gag+ cells, with respect to noninfected cells as described [33,40].

### Western blotting

For analysis of expression of auxiliary proteins in infected T cells, and APOBEC3G/A and MX2 in MDMs, cells (10^6^) were lysed in 100 μl of reducing Laemmli sample buffer (2x) supplemented with PhosSTOP (phosphatase inhibitor cocktail tablets; Sigma-Aldrich) and cOmplete (EDTA-free protease inhibitor cocktail; Sigma-Aldrich), and then boiled at 96°C for 10 min. Cell lysates were then resolved by SDS-PAGE as described [33], and analyzed by Western blotting using anti-Vif, anti-Nef, anti-Vpu, anti-Vpr, anti-HIV-1 p24 (Abcam; ab9071), anti-APOBEC3G/A (1/1000) and Mx2 (1/1000), and anti-β-actin (1/1000) antibodies.

### DNA-FISH analysis

After 24 h of coculture of NLAD8-infected Jurkat cells with female MDMs pretreated or not with antiretroviral drugs (AZT, NVP, and PF74) at the concentration of 10 mM, T cells were eliminated, and MDMs were analyzed by DNA-FISH performed as previously described [56]. Briefly, the HIV-1 NLAD8 (plasmid pNLAD8) and X-linked *KDM5C* gene (BAC clone RP11-236P24) probes were labeled by Nick translation (Vysis kit) with Aminoallyl-dUTP-ATTO-550 and Aminoallyl-dUTP-ATTO-488 respectively (Jena Bioscience). For one coverslip, around 200 ng of each probe were prepared separately: HIV-1 probe was precipitated, resuspended in 10 μl of hybridization buffer (50% Formamide, 20% Dextran sulfate, 2X SSC, 2 mg BSA) and denatured at 75°C for 7 min while *KDM5C* probe was precipitated with 1μg of human Cot-1 DNA, resuspended in 10 μl of hybridization buffer, denatured at 75°C for 7 min and incubated for 45 min at 37°C to anneal all the repetitive sequences. The two probes were then pooled just prior to overnight hybridization with the coverslips. MDMs cultured on coverslips were fixed in 2% paraformaldehyde for 15 min, permeabilized in 1X PBS/0.5% Triton X-100 on ice for 7 min, and progressively dehydrated in ethanol. Coverslips were then incubated in 0.1 μg/μl RNase A (Thermofisher) in 1X PBS for 1h at 37°C. After two washes in 1X PBS, cells were then incubated in ice-cold 0.7%-Triton/0.1M HCl for 10 min on ice, rinsed twice in 2X SSC and denatured in 50% Formamide/2X SSC for 30 min at 80°C. After 3 washes in ice-cold 2X SSC, cells were then hybridized with probes overnight at 37°C in a dark and humid chamber. After 3 washes in 50% formamide/2X SSC, 3 washes in 2X SSC at 42°C, and DAPI counterstaining, coverslips were mounted on slides and visualized under fluorescence microscope. Images were acquired on a spinning disk (CSU-X1M1; Yokogawa)-equipped inverted microscope (DMI6000; Leica) and were then processed using Fiji software (ImageJ; NIH). Quantitative image analysis and determination of HIV+ nuclei were analyzed from images of at least 30 MGCs followed by processing using Fiji and Procreate illustrate specific signals found in nuclei of representative MGCs.

### Statistical analysis

Statistics and curve fitting were performed using the GraphPad prism software. As mentioned in the figure legends, when comparisons were performed between more than 2 groups, ANOVA tests were used for statistical analyses, and *P* values were obtained by Dunnett’s post-tests correction to compare every mean to the control group mean. For all our experiments, graphics represent the results of at least three independent experiments (n = 3). Since each experiment was conducted using primary MDMs from different donors, the results for each experiment were normalized to the control group.

## Supporting information

S1 Fig Influence of HIV-1 auxiliary proteins in virus cell-free and cell-to-cell infection of macrophages. (A and B) MDMs were infected by the indicated cell-free viruses (CF) or cocultured for 24 h with Jurkat cells infected with the indicated viruses (CTC), and viral production (p24) was analyzed 6 days later. In B), are the results of a representative experiment performed in triplicate by coculture of infected Jurkat cells with MDMs from a representative donor. (C and D) MDMs were infected with the indicated cell-free (CF) viruses and analyzed by flow cytometry after intracellular Gag staining, after 6 h of infection (C) or 4 days post-infection (D). Error bars represent 1 SEM. Statistical significance was determined using the Mann-Whitney U-test (in A and B), and the Anova test (in C and D), and *P* values were obtained by Dunnett’s post-test correction (*P < 0.05; **P < 0.01; ***, *P* < 0.001; ****P < 0.0001).
(PDF)

S2 Fig Influence of HIV-1 auxiliary proteins on MGC formation. Jurkat T cells were infected with cell-free WT or mutated NLAD8 viruses and then cocultured for 24 h with MDMs. After elimination of T cells, MDMs were stained immediately after coculture (A) or 4 (B) and 6 (C) days later, with anti-Gag (green) antibodies, phalloidin (F-actin, red), and the nuclei were stained with Dapi (blue), before observation by confocal microscopy. These images correspond to the individual staining of the representative images shown in Fig 1. Scale bars are indicated. (D-F) Results are expressed as the percentage of Gag + MGCs with 2, 3, 4 or more than 4 nuclei quantified from a representative experiment (upper panels). Lower panels represent the levels of MDM infection (infection index), and are means of at least 4 independent experiments performed with MDMs of 4 different donors. Error bars represent 1 SEM. Statistical significance was determined using the Anova test, and P values were obtained by Dunnett’s post-test correction (ns, P > 0.05).
(PDF)

S3 Fig Influence of Nef on HIV-1 cell-to-cell transfer to macrophages, and MGC formation. Jurkat T cells were infected with WT or ΔNef viruses derived from the NLAD8 or 89.6 viral strains, and then cocultured for 24 h with MDMs before elimination of T cells. (A and B) MDMs were analyzed by flow cytometry after intracellular Gag staining just after the 24 h-coculture or 6 days after elimination of T cells. Results are expressed as the percentages of Gag + MDMs relative to that determined by coculture of MDMs with Jurkat cells infected with WT NLAD8 (A) or 89.6 (B) viruses (100%). Each dot corresponds to one donor. The results represent the means of at least 4 independent experiments performed with MDMs of at least 4 different donors. Error bars represent 1 SEM. Statistical significance was determined using the One-way Anova test (ns, P > 0.05; ****P < 0.0001). (C-J) MDMs were analyzed by confocal microscopy immediately after the 24h-coculture (C-F) or 6 days after elimination of T cells (G-J) with anti-Gag (green) antibodies, phalloidin (F-actin, red), and Dapi (blue). The total number of nuclei (Dapi+) per Gag + MDM was quantified on at least 100 cells. Representative images with scale bars are shown in (C, E G and I). In D, F, H and J), results are expressed as the percentage of Gag + MGCs with 1, 2, 3, 4 or more than 4 nuclei quantified from a representative experiment (right panels). In left panels), results are expressed as the means of total nucleus number per Gag + MGC and the results represent the means of at least 4 independent experiments performed with MDMs of 4 different donors. (NI), MDMs cocultured with non-infected Jurkat cells. Error bars represent 1 SEM. Statistical significance was determined using the Anova test, and P values were obtained by Dunnett’s post-test correction (ns, (ns, P > 0.05; ***, P < 0.001; ****P < 0.0001).
(PDF)

S4 Fig Infection of MDMs by cell-cell fusion with SERINC5-deleted Jurkat cells infected with WT viruses. (A and B) Parental (mock) or SERINC5-KO Jurkat cells (clones B1, B5, and F6) were infected with WT or Nef-deleted NLAD8 (A) or 89.6 (B) viruses, and analyzed for intracellular Gag expression by flow cytometry 36 h later. Results are the means of at least 6 independent experiments performed in duplicate. (C and D) Parental (mock) or SERINC5-KO Jurkat cells infected with WT NLAD8 (C) or 89.6 (D) viruses were cocultured for 24 h with MDMs. After elimination of T cells, MDMs were analyzed immediately (24 h) or cultured for 6 additional days before analysis by flow cytometry after intracellular Gag staining. Results are the means of at least 6 independent experiments performed with MDMs from at least 6 different donors, and are expressed as the percentages of Gag + MDMs relative to those determined after coculture of MDMs with the parental infected Jurkat cells (100%). Error bars represent 1 SEM. Statistical significance was determined using the One-way Anova test (ns, P > 0.05; **, P < 0.01). (E-L) MDMs were cocultured for 24 h with parental or SERINC5-KO Jurkat cells infected with the WT viruses, and then stained just after coculture or 6 days later with anti-Gag (green) antibodies, phalloidin (F-actin, red), while the nuclei were stained with Dapi (blue), before observation by confocal microscopy. The total number of nuclei (Dapi+) per Gag + MDM was quantified on at least 100 cells Representative images are shown in E, G, I and J), and scale bars are indicated. In F, H, K and L), results are expressed as the percentage of Gag + MDMs with 2, 3, 4 or more than 4 nuclei quantified from a representative experiment (right panels). In left panels), results are expressed as the means of total nucleus number per Gag + MDM, and represent the means of at least 4 independent experiments performed with MDMs of 4 different donors. Error bars represent 1 SEM. Statistical significance was determined using the Anova test, and P values were obtained by Dunnett’s post-test correction (ns, P > 0.05; *, P < 0.05).
(PDF)

S5 Fig IFN-I activity on HIV-1 cell-free and cell-to-cell infection of macrophages. MDMs were inoculated with cell-free viruses (CF), or cocultured (CTC) with autologous infected T cells for 6 h in the absence (mock) or presence of the indicated concentration of IFNa. After elimination of the virus inoculum or infected T cells, MDMs were analyzed just after the 6 h of coculture, or cultured for 6 additional days in the absence (mock) or presence of the indicated concentration of IFNa. (A and E) flow cytometry analysis. Results are expressed as the percentage of Gag + MDMs relative to that measured without IFN, after 6 h of coculture (A) or 6 days later (E). (B-D and F-H) Results represent the quantitative analysis measured from the images of cell-free (CF) infection shown in Fig 3D, lower panels. In B and F), results are expressed as the percentage of Gag + MDMs with 1, 2, 3, 4 or more than 4 nuclei. In C and G), results correspond to the level of infection (infection index) quantified from images of cell-free infection of MDMs. In D and H), results are expressed as the means of nucleus number per Gag + , and represent the means of at least 4 independent experiments performed with MDMs of 4 different donors. Error bars represent 1 SEM. Statistical significance was determined using the Anova test, and *P* values were obtained by Dunnett’s post-test correction (ns, *P* > 0.05; **, *P* < 0.01; ****, *P* < 0.0001). In I), individual staining of the representative images shown in Fig 3D. In S5J and S5K), results are expressed as the percentage of Gag + MDMs with 2, 3, 4 or more than 4 nuclei. In S5L and S5M), results correspond to the level of infection (infection index) quantified from images.
(PDF)

S6 Fig Role of APOBEC3G in HIV-1 cell-to-cell infection of macrophages by cell-cell fusion with infected T cells. (A) Experimental design, created with Biorender, for depletion of APOBEC3G with siRNA (siA3G) before treatment of MDMs with IFNα (1,000 U/mL), and virus cell-to-cell spreading from infected T cells in the presence of IFNα. After elimination of T cells, MDMs were analyzed immediately or after 6 additional days in culture by flow cytometry or confocal microscopy. In B and C) are shown the individual staining corresponding to the images shown in Fig 3N.
(PDF)

S7 Fig Transcriptional activity of the Jurkat T cell nuclei in MGCs formed upon cell-cell fusion. (A) Jurkat-LTR-GFP cells were infected with the HIV-1 NLAD8 strains, and analyzed 36 h later by flow cytometry after intracellular Gag staining. The results correspond to the percentage of Gag + T cells expressing GFP (green bar) or not (blue bar), and are the means of 4 independent experiments performed in duplicate. (B) Infected Jurkat-LTR-GFP cells were cocultured with MDMs for 24 h. After elimination of T cells, MDMs were then cultured for different period of time (from D0 to D20) before analysis by flow cytometry of the level of Gag-positive cells. The results correspond to the means of at least 4 independent experiments performed on at least 4 different donors. Error bars represent 1 SEM. (C-J) Infected Jurkat-LTR-GFP were cocultured for 24 h with MDMs. T cells were then eliminated, and the MDMs were cultured for 12 (C-F) or 20 (G-J) additional days before analysis by fluorescence microscopy after staining with anti-Gag (brown yellow) antibodies, phalloidin (F-actin, red), Dapi (Nucleus, blue) and GFP (green). Representative images are shown in C) and G), and the total number of nuclei (Dapi+) per Gag + MDM was quantified on at least 100 cells. In D and H), the results are the percentage of Gag-positive MGCs co-expressing GFP (green bar) or not (blue bar). In E and I), results are expressed as the percentage of Gag + MDMs with 1, 2, 3, or more than 3 nuclei in GFP-negative (central bar) and -positive cells (right bar). In F and J), results are the mean of nuclei per Gag + MGCs in co-expressing GFP cells (left green bar) or not (central blue bar). NI, MDMs cocultured for 24 h with noninfected Jurkat-LTR-GFP cells, and then cultured for 20 additional days before fluorescence microscopy.
(PDF)

S8 Fig Confocal microscopy images of HIV-1 cell-free and cell-to-cell infection of MDMs in the presence of antiretroviral drugs. MDMs pretreated with the indicated antiretroviral drugs (e.i., PF74, AZT, NVP, or RAL) were infected by cell-free NLAD8 viruses or cocultured for 24 h with NLAD8-infected Jurkat cells. MDMs were then stained with anti-Gag (green) antibodies, phalloidin (F-actin, red), and Dapi (Nucleus, blue), before observation by confocal microscopy. The images shown correspond to the individual staining of the representative images shown in Fig 5B. Scale bars are indicated. (A) Images of cell-to-cell infected MDMs analyzed just after the 24 h of coculture. (B) Images of cell-to-cell infected macrophages cultured for 4 days after the coculture and elimination of infected T cells. (C) Images of cell-free infected MDMs cultured for 4 days after elimination of the viral inoculum. (D) Results are expressed as the percentage of Gag + MDMs with 1, 2, 3, 4 or more than 4 nuclei quantified from the images shown in C) on at least 100 cells. (E) Results are the means of nuclei per MDM quantified from the images shown in C), and represent the means of at least 4 independent experiments performed with MDMs of 4 different donors. Error bars represent 1 SEM. Statistical significance was determined using the Anova test, and *P* values were obtained by Dunnett’s post-test correction (ns, P > 0.05; ****, P < 0.0001).
(PDF)

S9 Fig FISH analysis of HIV-1 DNA in lymphoid nuclei of MGCs. MDMs were cocultured for 6 or 24 h with non-infected- or NLAD8-infected Jurkat or primary CD4 T cells, or cultured for 4 additional days after coculture before DNA-FISH analysis. MDMs were fixed, permeabilized, stained with Dapi, and then incubated after RNAse treatment with specific probes for detection of the KDM5C gene located on the X chromosome together with the HIV-NLAD8 probe for detection of the proviral DNA. (A) Quantification of the percentage of MGCs with T cell nuclei containing HIV-positive dots after 6 or 24 h of coculture, or 4 days later from the representative experiment shown in Fig 6. The number of HIV-positive nuclei was quantified in at least 30 MGCs. (B and C) MDMs pretreated or not with antiretroviral drugs (PF74, AZT, nevirapine or raltegravir) were cocultured for 6 or 24 h with NLAD8-infected Jurkat (panel B) or primary CD4 T cells (panel C), or cultured after elimination of T cells for 4 additional days in the presence of the drugs before DNA-FISH analysis. MDMs were fixed, permeabilized, stained with Dapi, and then incubated with probes for detection of the KDM5C gene together with the HIV-NLAD8 probe. Representative images of infected multinucleated MDMs after 6 or 24 h of coculture, or 4 days later are shown (left, middle, and right images, respectively). Scale bar is indicated.
(PDF)

S1 File Confirmation of publication and licensing rights BioRender. (PDF)

## References

[1] Woottum M, Yan S, Sayettat S, Grinberg S, Cathelin D, Bekaddour N, et al. Macrophages: key cellular players in HIV infection and pathogenesis. Viruses. 2024;16(2):288. doi: 10.3390/v16020288 38400063 PMC10893316

[2] Chitrakar A, Sanz M, Maggirwar SB, Soriano-Sarabia N. HIV latency in myeloid cells: challenges for a cure. Pathogens. 2022;11(6):611. doi: 10.3390/pathogens11060611 35745465 PMC9230125

[3] Vine EE, Rhodes JW, Warner van Dijk FA, Byrne SN, Bertram KM, Cunningham AL, et al. HIV transmitting mononuclear phagocytes; integrating the old and new. Mucosal Immunol. 2022;15(4):542–50. doi: 10.1038/s41385-022-00492-0 35173293 PMC9259493

[4] Hendricks CM, Cordeiro T, Gomes AP, Stevenson M. The interplay of HIV-1 and macrophages in viral persistence. Front Microbiol. 2021;12:646447. doi: 10.3389/fmicb.2021.646447 33897659 PMC8058371

[5] Kruize Z, Kootstra NA. The role of macrophages in HIV-1 persistence and pathogenesis. Front Microbiol. 2019;10:2828. doi: 10.3389/fmicb.2019.02828 31866988 PMC6906147

[6] Ganor Y, Real F, Sennepin A, Dutertre C-A, Prevedel L, Xu L, et al. HIV-1 reservoirs in urethral macrophages of patients under suppressive antiretroviral therapy. Nat Microbiol. 2019;4(4):633–44. doi: 10.1038/s41564-018-0335-z 30718846

[7] Rodrigues V, Ruffin N, San-Roman M, Benaroch P. Myeloid cell interaction with HIV: a complex relationship. Front Immunol. 2017;8:1698. doi: 10.3389/fimmu.2017.01698 29250073 PMC5714857

[8] Veenhuis RT, Abreu CM, Costa PAG, Ferreira EA, Ratliff J, Pohlenz L, et al. Monocyte-derived macrophages contain persistent latent HIV reservoirs. Nat Microbiol. 2023;8(5):833–44. doi: 10.1038/s41564-023-01349-3 36973419 PMC10159852

[9] Tang Y, Chaillon A, Gianella S, Wong LM, Li D, Simermeyer TL, et al. Brain microglia serve as a persistent HIV reservoir despite durable antiretroviral therapy. J Clin Invest. 2023;133(12):e167417. doi: 10.1172/JCI167417 37317962 PMC10266791

[10] Moar P, Premeaux TA, Atkins A, Ndhlovu LC. The latent HIV reservoir: current advances in genetic sequencing approaches. mBio. 2023;14(5):e0134423. doi: 10.1128/mbio.01344-23 37811964 PMC10653892

[11] Joseph J, Daley W, Lawrence D, Lorenzo E, Perrin P, Rao VR, et al. Role of macrophages in HIV pathogenesis and cure: NIH perspectives. J Leukoc Biol. 2022;112(5):1233–43. doi: 10.1002/JLB.4MR0722-619R 36073341

[12] Andrade VM, Mavian C, Babic D, Cordeiro T, Sharkey M, Barrios L, et al. A minor population of macrophage-tropic HIV-1 variants is identified in recrudescing viremia following analytic treatment interruption. Proc Natl Acad Sci U S A. 2020;117(18):9981–90. doi: 10.1073/pnas.1917034117 32300019 PMC7211992

[13] Wong ME, Jaworowski A, Hearps AC. The HIV reservoir in monocytes and macrophages. Front Immunol. 2019;10:1435. doi: 10.3389/fimmu.2019.01435 31297114 PMC6607932

[14] Burdo TH, Lackner A, Williams KC. Monocyte/macrophages and their role in HIV neuropathogenesis. Immunol Rev. 2013;254(1):102–13. doi: 10.1111/imr.12068 23772617 PMC3704190

[15] Carter CA, Ehrlich LS. Cell biology of HIV-1 infection of macrophages. Annu Rev Microbiol. 2008;62:425–43. doi: 10.1146/annurev.micro.62.081307.162758 18785842

[16] Costiniuk CT, Jenabian M-A. The lungs as anatomical reservoirs of HIV infection. Rev Med Virol. 2014;24(1):35–54. doi: 10.1002/rmv.1772 24151040

[17] Dargent JL, Lespagnard L, Kornreich A, Hermans P, Clumeck N, Verhest A. HIV-associated multinucleated giant cells in lymphoid tissue of the Waldeyer’s ring: a detailed study. Mod Pathol. 2000;13(12):1293–9. doi: 10.1038/modpathol.3880237 11144925

[18] Frankel SS, Wenig BM, Burke AP, Mannan P, Thompson LD, Abbondanzo SL, et al. Replication of HIV-1 in dendritic cell-derived syncytia at the mucosal surface of the adenoid. Science. 1996;272(5258):115–7. doi: 10.1126/science.272.5258.115 8600520

[19] Harbison C, Zhuang K, Gettie A, Blanchard J, Knight H, Didier P, et al. Giant cell encephalitis and microglial infection with mucosally transmitted simian-human immunodeficiency virus SHIVSF162P3N in rhesus macaques. J Neurovirol. 2014;20(1):62–72. doi: 10.1007/s13365-013-0229-z 24464410 PMC4337388

[20] Matsuda K, Riddick NE, Lee CA, Puryear SB, Wu F, Lafont BAP, et al. A SIV molecular clone that targets the CNS and induces neuroAIDS in rhesus macaques. PLoS Pathog. 2017;13(8):e1006538. doi: 10.1371/journal.ppat.1006538 28787449 PMC5560746

[21] Soulas C, Conerly C, Kim W-K, Burdo TH, Alvarez X, Lackner AA, et al. Recently infiltrating MAC387(+) monocytes/macrophages a third macrophage population involved in SIV and HIV encephalitic lesion formation. Am J Pathol. 2011;178(5):2121–35. doi: 10.1016/j.ajpath.2011.01.023 21514427 PMC3081227

[22] Teo I, Veryard C, Barnes H, An SF, Jones M, Lantos PL, et al. Circular forms of unintegrated human immunodeficiency virus type 1 DNA and high levels of viral protein expression: association with dementia and multinucleated giant cells in the brains of patients with AIDS. J Virol. 1997;71(4):2928–33. doi: 10.1128/JVI.71.4.2928-2933.1997 9060651 PMC191420

[23] Vicandi B, Jiménez-Heffernan JA, López-Ferrer P, Patrón M, Gamallo C, Colmenero C, et al. HIV-1 (p24)-positive multinucleated giant cells in HIV-associated lymphoepithelial lesion of the parotid gland. A report of two cases. Acta Cytol. 1999;43(2):247–51. doi: 10.1159/000330987 10097719

[24] Watters SA, Mlcochova P, Gupta RK. Macrophages: the neglected barrier to eradication. Curr Opin Infect Dis. 2013;26(6):561–6. doi: 10.1097/QCO.0000000000000014 24152764

[25] Honeycutt JB, Thayer WO, Baker CE, Ribeiro RM, Lada SM, Cao Y, et al. HIV persistence in tissue macrophages of humanized myeloid-only mice during antiretroviral therapy. Nat Med. 2017;23(5):638–43. doi: 10.1038/nm.4319 28414330 PMC5419854

[26] Zhang C, Zaman LA, Poluektova LY, Gorantla S, Gendelman HE, Dash PK. Humanized mice for studies of HIV-1 persistence and elimination. Pathogens. 2023;12(7):879. doi: 10.3390/pathogens12070879 37513726 PMC10383313

[27] Araínga M, Edagwa B, Mosley RL, Poluektova LY, Gorantla S, Gendelman HE. A mature macrophage is a principal HIV-1 cellular reservoir in humanized mice after treatment with long acting antiretroviral therapy. Retrovirology. 2017;14(1):17. doi: 10.1186/s12977-017-0344-7 28279181 PMC5345240

[28] Mathews S, Branch Woods A, Katano I, Makarov E, Thomas MB, Gendelman HE, et al. Human Interleukin-34 facilitates microglia-like cell differentiation and persistent HIV-1 infection in humanized mice. Mol Neurodegener. 2019;14(1):12. doi: 10.1186/s13024-019-0311-y 30832693 PMC6399898

[29] Han M, Woottum M, Mascarau R, Vahlas Z, Verollet C, Benichou S. Mechanisms of HIV-1 cell-to-cell transfer to myeloid cells. J Leukoc Biol. 2022;112(5):1261–71. doi: 10.1002/JLB.4MR0322-737R 35355323

[30] Dupont M, Sattentau QJ. Macrophage cell-cell interactions promoting HIV-1 infection. Viruses. 2020;12(5):492. doi: 10.3390/v12050492 32354203 PMC7290394

[31] Swanstrom R, Coffin J. HIV-1 pathogenesis: the virus. Cold Spring Harb Perspect Med. 2012;2(12):a007443. doi: 10.1101/cshperspect.a007443 23143844 PMC3543077

[32] Bowen NE, Oo A, Kim B. Mechanistic interplay between HIV-1 reverse transcriptase enzyme kinetics and host SAMHD1 protein: viral myeloid-cell tropism and genomic mutagenesis. Viruses. 2022;14(8):1622. doi: 10.3390/v14081622 35893688 PMC9331428

[33] Xie M, Leroy H, Mascarau R, Woottum M, Dupont M, Ciccone C, et al. Cell-to-cell spreading of HIV-1 in myeloid target cells escapes SAMHD1 restriction. mBio. 2019;10(6):e02457-19. doi: 10.1128/mBio.02457-19 31744918 PMC6867896

[34] Colomer-Lluch M, Ruiz A, Moris A, Prado JG. Restriction factors: from intrinsic viral restriction to shaping cellular immunity against HIV-1. Front Immunol. 2018;9:2876. doi: 10.3389/fimmu.2018.02876 30574147 PMC6291751

[35] Cribier A, Descours B, Valadão ALC, Laguette N, Benkirane M. Phosphorylation of SAMHD1 by cyclin A2/CDK1 regulates its restriction activity toward HIV-1. Cell Rep. 2013;3(4):1036–43. doi: 10.1016/j.celrep.2013.03.017 23602554

[36] Herate C, Vigne C, Guenzel CA, Lambele M, Rouyez M-C, Benichou S. Uracil DNA glycosylase interacts with the p32 subunit of the replication protein A complex to modulate HIV-1 reverse transcription for optimal virus dissemination. Retrovirology. 2016;13:26. doi: 10.1186/s12977-016-0257-x 27068393 PMC4828845

[37] Pagani I, Demela P, Ghezzi S, Vicenzi E, Pizzato M, Poli G. Host restriction factors modulating HIV latency and replication in macrophages. Int J Mol Sci. 2022;23(6):3021. doi: 10.3390/ijms23063021 35328442 PMC8951319

[38] Volcic M, Wiesmüller L, Kirchhoff F. Small but highly versatile: the viral accessory protein Vpu. Annu Rev Virol. 2023;10(1):243–59. doi: 10.1146/annurev-virology-111821-100816 37406340

[39] Bracq L, Xie M, Benichou S, Bouchet J. Mechanisms for cell-to-cell transmission of HIV-1. Front Immunol. 2018;9:260. doi: 10.3389/fimmu.2018.00260 29515578 PMC5825902

[40] Bracq L, Xie M, Lambelé M, Vu L-T, Matz J, Schmitt A, et al. T cell-macrophage fusion triggers multinucleated giant cell formation for HIV-1 spreading. J Virol. 2017;91(24):e01237-17. doi: 10.1128/JVI.01237-17 28978713 PMC5709600

[41] Raynaud-Messina B, Bracq L, Dupont M, Souriant S, Usmani SM, Proag A, et al. Bone degradation machinery of osteoclasts: an HIV-1 target that contributes to bone loss. Proc Natl Acad Sci U S A. 2018;115(11):E2556–65. doi: 10.1073/pnas.1713370115 29463701 PMC5856515

[42] Han M, Cantaloube-Ferrieu V, Xie M, Armani-Tourret M, Woottum M, Pagès J-C, et al. HIV-1 cell-to-cell spread overcomes the virus entry block of non-macrophage-tropic strains in macrophages. PLoS Pathog. 2022;18(5):e1010335. doi: 10.1371/journal.ppat.1010335 35622876 PMC9182568

[43] Mascarau R, Woottum M, Fromont L, Gence R, Cantaloube-Ferrieu V, Vahlas Z, et al. Productive HIV-1 infection of tissue macrophages by fusion with infected CD4+ T cells. J Cell Biol. 2023;222(5):e202205103. doi: 10.1083/jcb.202205103 36988579 PMC10067447

[44] Harms M, Smith N, Han M, Groß R, von Maltitz P, Stürzel C, et al. Spermine and spermidine bind CXCR4 and inhibit CXCR4- but not CCR5-tropic HIV-1 infection. Sci Adv. 2023;9(27):eadf8251. doi: 10.1126/sciadv.adf8251 37406129 PMC10321752

[45] Xu S, Zheng Z, Pathak JL, Cheng H, Zhou Z, Chen Y, et al. The emerging role of the serine incorporator protein family in regulating viral infection. Front Cell Dev Biol. 2022;10:856468. doi: 10.3389/fcell.2022.856468 35433679 PMC9010877

[46] Leonhardt SA, Purdy MD, Grover JR, Yang Z, Poulos S, McIntire WE, et al. Antiviral HIV-1 SERINC restriction factors disrupt virus membrane asymmetry. Nat Commun. 2023;14(1):4368. doi: 10.1038/s41467-023-39262-2 37474505 PMC10359404

[47] Passos V, Zillinger T, Casartelli N, Wachs AS, Xu S, Malassa A, et al. Characterization of endogenous SERINC5 protein as anti-HIV-1 factor. J Virol. 2019;93(24):e01221-19. doi: 10.1128/JVI.01221-19 31597782 PMC6880170

[48] Beitari S, Ding S, Pan Q, Finzi A, Liang C. Effect of HIV-1 Env on SERINC5 antagonism. J Virol. 2017;91(4):e02214-16. doi: 10.1128/JVI.02214-16 27928004 PMC5286904

[49] Stavrou S, Ross SR. APOBEC3 proteins in viral immunity. J Immunol. 2015;195(10):4565–70. doi: 10.4049/jimmunol.1501504 26546688 PMC4638160

[50] Staeheli P, Haller O. Human MX2/MxB: a potent interferon-induced postentry inhibitor of herpesviruses and HIV-1. J Virol. 2018;92(24):e00709-18. doi: 10.1128/JVI.00709-18 30258007 PMC6258936

[51] Ikeda T, Molan AM, Jarvis MC, Carpenter MA, Salamango DJ, Brown WL, et al. HIV-1 restriction by endogenous APOBEC3G in the myeloid cell line THP-1. J Gen Virol. 2019;100(7):1140–52. doi: 10.1099/jgv.0.001276 31145054 PMC7176283

[52] Chaipan C, Smith JL, Hu W-S, Pathak VK. APOBEC3G restricts HIV-1 to a greater extent than APOBEC3F and APOBEC3DE in human primary CD4+ T cells and macrophages. J Virol. 2013;87(1):444–53. doi: 10.1128/JVI.00676-12 23097438 PMC3536366

[53] Rankovic S, Ramalho R, Aiken C, Rousso I. PF74 reinforces the HIV-1 capsid to impair reverse transcription-induced uncoating. J Virol. 2018;92(20):e00845-18. doi: 10.1128/JVI.00845-18 30089694 PMC6158434

[54] Balasubramaniam M, Pandhare J, Dash C. Immune control of HIV. J Life Sci (Westlake Village). 2019;1(1):4–37. 31468033 PMC6714987

[55] Gillis S, Watson J. Biochemical and biological characterization of lymphocyte regulatory molecules. V. Identification of an interleukin 2-producing human leukemia T cell line. J Exp Med. 1980;152(6):1709–19. doi: 10.1084/jem.152.6.1709 6778951 PMC2186024

[56] Chaumeil J, Micsinai M, Skok JA. Combined immunofluorescence and DNA FISH on 3D-preserved interphase nuclei to study changes in 3D nuclear organization. J Vis Exp. 2013;(72):e50087. doi: 10.3791/50087 23407477 PMC3597039

[57] Calantone N, Wu F, Klase Z, Deleage C, Perkins M, Matsuda K, et al. Tissue myeloid cells in SIV-infected primates acquire viral DNA through phagocytosis of infected T cells. Immunity. 2014;41(3):493–502. doi: 10.1016/j.immuni.2014.08.014 25238099 PMC4241569

[58] DiNapoli SR, Ortiz AM, Wu F, Matsuda K, Twigg HL 3rd, Hirsch VM, et al. Tissue-resident macrophages can contain replication-competent virus in antiretroviral-naive, SIV-infected Asian macaques. JCI Insight. 2017;2(4):e91214. doi: 10.1172/jci.insight.91214 28239657 PMC5313072

[59] Guenzel CA, Hérate C, Benichou S. HIV-1 Vpr-a still “enigmatic multitasker”. Front Microbiol. 2014;5:127. doi: 10.3389/fmicb.2014.00127 24744753 PMC3978352

[60] Vérollet C, Zhang YM, Le Cabec V, Mazzolini J, Charrière G, Labrousse A, et al. HIV-1 Nef triggers macrophage fusion in a p61Hck- and protease-dependent manner. J Immunol. 2010;184(12):7030–9. doi: 10.4049/jimmunol.0903345 20488787

[61] Vérollet C, Le Cabec V, Maridonneau-Parini I. HIV-1 infection of T lymphocytes and macrophages affects their migration via Nef. Front Immunol. 2015;6:514. doi: 10.3389/fimmu.2015.00514 26500651 PMC4594015

[62] Mazzolini J, Herit F, Bouchet J, Benmerah A, Benichou S, Niedergang F. Inhibition of phagocytosis in HIV-1-infected macrophages relies on Nef-dependent alteration of focal delivery of recycling compartments. Blood. 2010;115(21):4226–36. doi: 10.1182/blood-2009-12-259473 20299515

[63] Cong L, Sugden SM, Leclair P, Lim CJ, Pham TNQ, Cohen ÉA. HIV-1 Vpu promotes phagocytosis of infected CD4+ T cells by macrophages through downregulation of CD47. mBio. 2021;12(4):e0192021. doi: 10.1128/mBio.01920-21 34425695 PMC8406190

[64] Baxter AE, Russell RA, Duncan CJA, Moore MD, Willberg CB, Pablos JL, et al. Macrophage infection via selective capture of HIV-1-infected CD4+ T cells. Cell Host Microbe. 2014;16(6):711–21. doi: 10.1016/j.chom.2014.10.010 25467409 PMC4271767

[65] Ramirez PW, Vollbrecht T, Acosta FM, Suarez M, Angerstein AO, Wallace J, et al. Nef enhances HIV-1 replication and infectivity independently of SERINC5 in CEM T cells. Virology. 2023;578:154–62. doi: 10.1016/j.virol.2022.12.008 36577173 PMC10484624

[66] Rosa A, Chande A, Ziglio S, De Sanctis V, Bertorelli R, Goh SL, et al. HIV-1 Nef promotes infection by excluding SERINC5 from virion incorporation. Nature. 2015;526(7572):212–7. doi: 10.1038/nature15399 26416734 PMC4861059

[67] Usami Y, Wu Y, Göttlinger HG. SERINC3 and SERINC5 restrict HIV-1 infectivity and are counteracted by Nef. Nature. 2015;526(7572):218–23. doi: 10.1038/nature15400 26416733 PMC4600458

[68] Pierini V, Gallucci L, Stürzel CM, Kirchhoff F, Fackler OT. SERINC5 can enhance proinflammatory cytokine production by primary human myeloid cells in response to challenge with HIV-1 particles. J Virol. 2021;95(9):e02372-20. doi: 10.1128/JVI.02372-20 33597208 PMC8104095

[69] McNab FW, Ewbank J, Howes A, Moreira-Teixeira L, Martirosyan A, Ghilardi N, et al. Type I IFN induces IL-10 production in an IL-27-independent manner and blocks responsiveness to IFN-γ for production of IL-12 and bacterial killing in Mycobacterium tuberculosis-infected macrophages. J Immunol. 2014;193(7):3600–12. doi: 10.4049/jimmunol.1401088 25187652 PMC4170673

[70] Xu S, Ducroux A, Ponnurangam A, Vieyres G, Franz S, Müsken M, et al. cGAS-mediated innate immunity spreads intercellularly through HIV-1 Env-induced membrane fusion sites. Cell Host Microbe. 2016;20(4):443–57. doi: 10.1016/j.chom.2016.09.003 27736643

[71] Ikeda T, Shimizu R, Nasser H, Carpenter MA, Cheng AZ, Brown WL, et al. APOBEC3 degradation is the primary function of HIV-1 Vif determining virion infectivity in the myeloid cell line THP-1. mBio. 2023;14(4):e0078223. doi: 10.1128/mbio.00782-23 37555667 PMC10470580

[72] Stultz RD, Cenker JJ, McDonald D. Imaging HIV-1 genomic DNA from entry through productive infection. J Virol. 2017;91(9):e00034-17. doi: 10.1128/JVI.00034-17 28250118 PMC5391475

[73] Francis AC, Marin M, Singh PK, Achuthan V, Prellberg MJ, Palermino-Rowland K, et al. HIV-1 replication complexes accumulate in nuclear speckles and integrate into speckle-associated genomic domains. Nat Commun. 2020;11(1). doi: 10.1038/s41467-020-17256-8 PMC7360574 32665593

[74] Rensen E, Mueller F, Scoca V, Parmar JJ, Souque P, Zimmer C, et al. Clustering and reverse transcription of HIV-1 genomes in nuclear niches of macrophages. EMBO J. 2021;40(1):e105247. doi: 10.15252/embj.2020105247 33270250 PMC7780146

[75] Bejarano DA, Peng K, Laketa V, Börner K, Jost KL, Lucic B, et al. HIV-1 nuclear import in macrophages is regulated by CPSF6-capsid interactions at the nuclear pore complex. Elife. 2019;8:e41800. doi: 10.7554/eLife.41800 30672737 PMC6400501

[76] Müller TG, Zila V, Müller B, Kräusslich H-G. Nuclear capsid uncoating and reverse transcription of HIV-1. Annu Rev Virol. 2022;9(1):261–84. doi: 10.1146/annurev-virology-020922-110929 35704745

[77] Compton AA, Schwartz O. They might be giants: Does syncytium formation sink or spread HIV infection?. PLoS Pathog. 2017;13(2):e1006099. doi: 10.1371/journal.ppat.1006099 28152024 PMC5289631

[78] Leroy H, Han M, Woottum M, Bracq L, Bouchet J, Xie M, et al. Virus-mediated cell-cell fusion. Int J Mol Sci. 2020;21(24):9644. doi: 10.3390/ijms21249644 33348900 PMC7767094

