# Supplementary material for: HIV-1 cell-to-cell infection of macrophages escapes type I interferon and host restriction factors, and is resistant to antiretroviral drugs
Source: PLoS Pathog. 2025 Apr 28;21(4):e1013130. doi: 10.1371/journal.ppat.1013130 (PMC12064042; doi:10.1371/journal.ppat.1013130)
Supplement: S1 Fig — (A and B) MDMs were infected by the indicated cell-free viruses (CF) or cocultured for 24 h with Jurkat cells infected with the indicated viruses (CTC), and viral production (p24) was analyzed 6 days later. In B), are the results of a representative experiment performed in triplicate by coculture of infected Jurkat cells with MDMs from a representative donor. (C and D) MDMs were infected with the indicated cell-free (CF) viruses and analyzed by flow cytometry after intracellular Gag staining, after 6 h of infection (C) or 4 days post-infection (D). Error bars represent 1 SEM. Statistical significance was determined using the Mann-Whitney U-test (in A and B), and the Anova test (in C and D), and P values were obtained by Dunnett’s post-test correction (*P < 0.05; **P < 0.01; ***, P < 0.001; ****P < 0.0001). (PDF) [file ppat.1013130.s001.pdf]

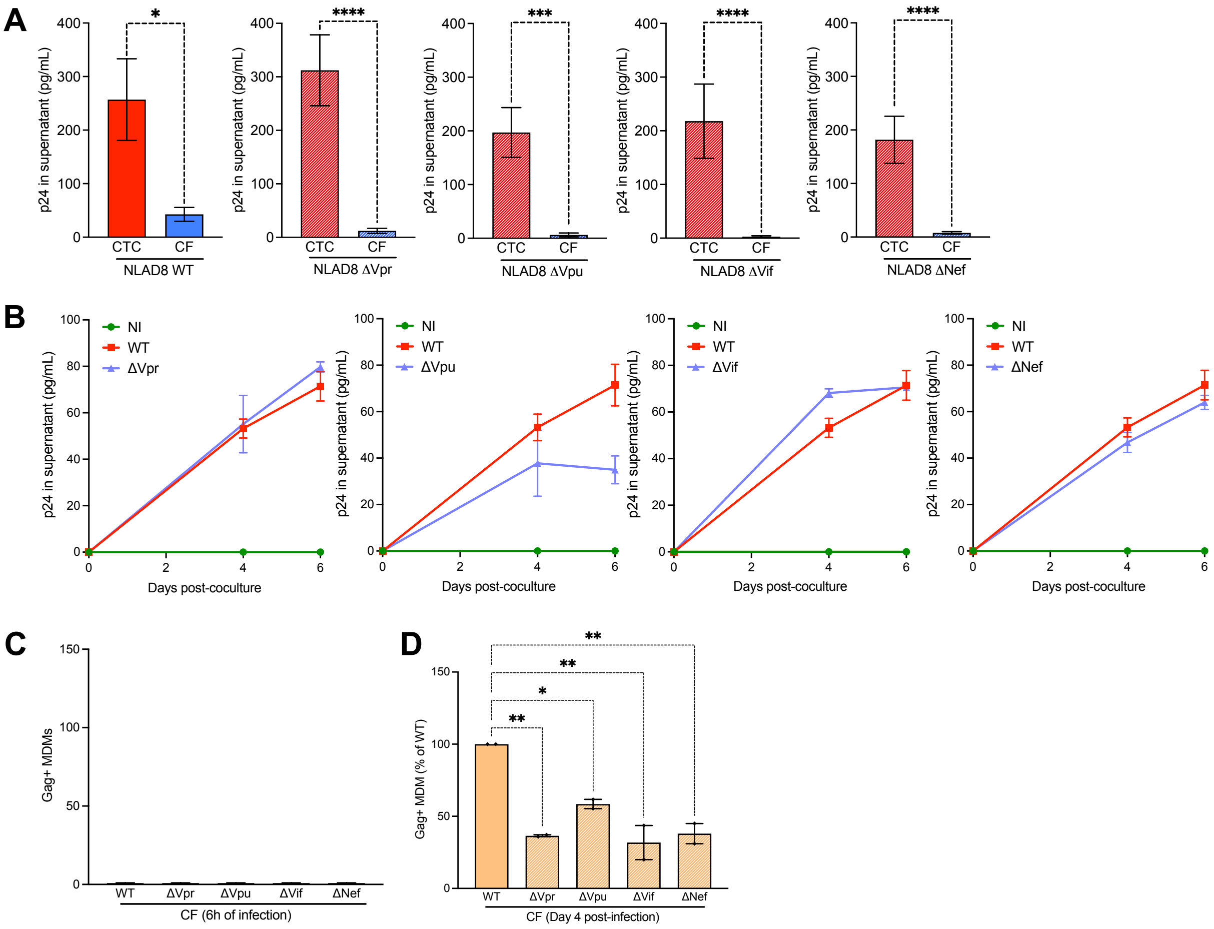


**S1 Fig. Influence of HIV-1 auxiliary proteins in virus cell-free and cell-to-cell infection of macrophages.** (A and B) MDMs were infected by the indicated cell-free viruses (CF) or cocultured for 24 h with Jurkat cells infected with the indicated viruses (CTC), and viral production (p24) was analyzed 6 days later. In B), are the results of a representative experiment performed in triplicate by coculture of infected Jurkat cells with MDMs from a representative donor. (C and D) MDMs were infected with the indicated cell-free (CF) viruses and analyzed by flow cytometry after intracellular Gag staining, after 6 h of infection (C) or 4 days post-infection (D). Error bars represent 1 SEM. Statistical significance was determined using the Mann-Whitney U-test (in A and B), and the Anova test (in C and D), and *P* values were obtained by Dunnett's post-test correction (**P*< 0.05; ***P*<0.01; ***, *P*<0.001; *****P*<0.0001).
