# Supplementary material for: HIV-1 cell-to-cell infection of macrophages escapes type I interferon and host restriction factors, and is resistant to antiretroviral drugs
Source: PLoS Pathog. 2025 Apr 28;21(4):e1013130. doi: 10.1371/journal.ppat.1013130 (PMC12064042; doi:10.1371/journal.ppat.1013130)
Supplement: S2 Fig — Jurkat T cells were infected with cell-free WT or mutated NLAD8 viruses and then cocultured for 24 h with MDMs. After elimination of T cells, MDMs were stained immediately after coculture (A) or 4 (B) and 6 (C) days later, with anti-Gag (green) antibodies, phalloidin (F-actin, red), and the nuclei were stained with Dapi (blue), before observation by confocal microscopy. These images correspond to the individual staining of the representative images shown in Fig 1. Scale bars are indicated. (D-F) Results are expressed as the percentage of Gag + MGCs with 2, 3, 4 or more than 4 nuclei quantified from a representative experiment (upper panels). Lower panels represent the levels of MDM infection (infection index), and are means of at least 4 independent experiments performed with MDMs of 4 different donors. Error bars represent 1 SEM. Statistical significance was determined using the Anova test, and P values were obtained by Dunnett’s post-test correction (ns, P > 0.05). (PDF) [file ppat.1013130.s002.pdf]

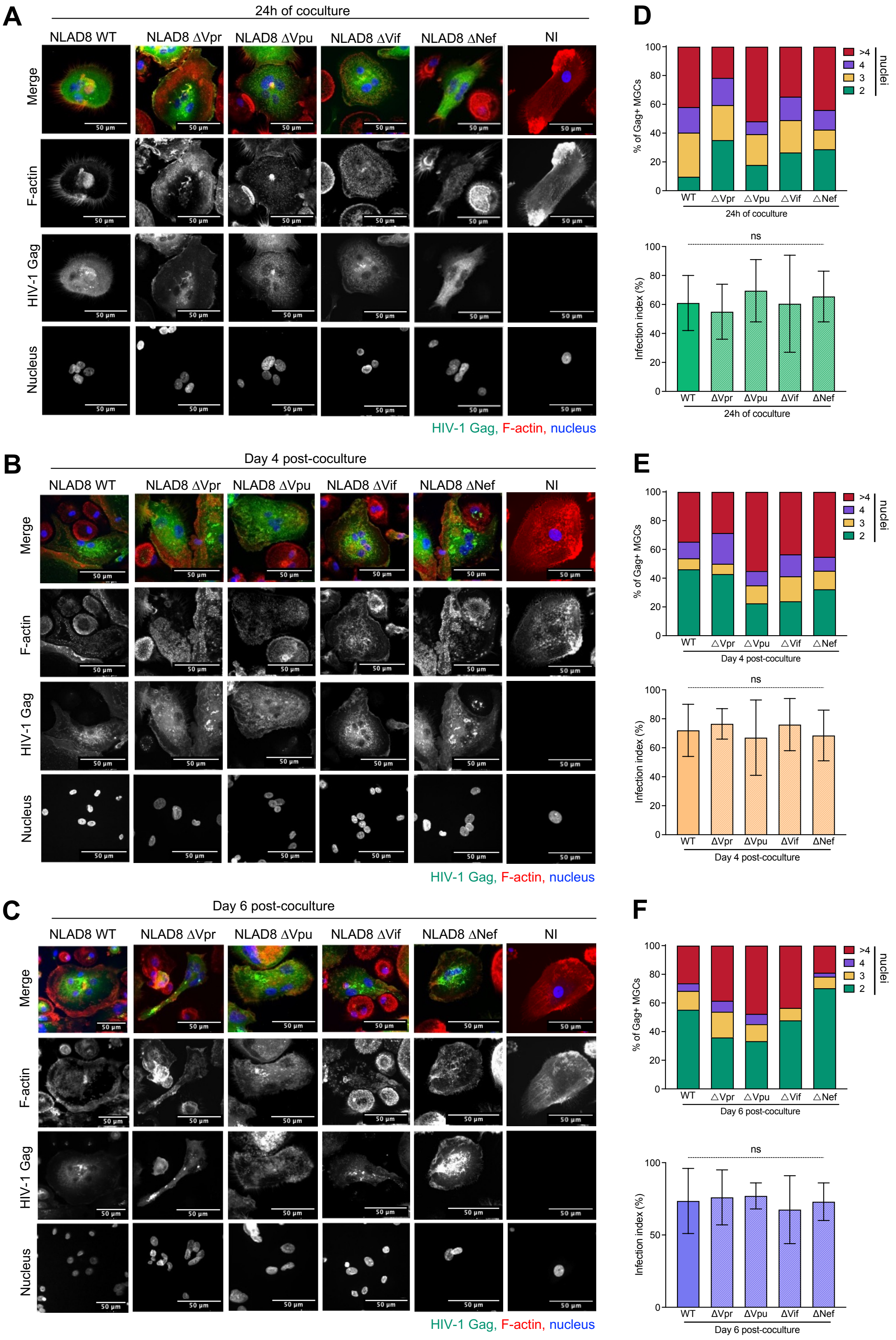

S2 Fig. Influence of HIV-1 auxiliary proteins on MGC formation. Jurkat T cells were infected with cell-free WT or mutated NLAD8 viruses and then cocultured for 24 h with MDMs. After elimination of T

cells, MDMs were stained immediately after coculture (A) or 4 (B) and 6 (C) days later, with anti-Gag (green) antibodies, phalloidin (F-actin, red), and the nuclei were stained with Dapi (blue), before observation by confocal microscopy. These images correspond to the individual staining of the representative images shown in Fig. 1. Scale bars are indicated. (D-F) Results are expressed as the percentage of Gag+ MGCs with 2, 3, 4 or more than 4 nuclei quantified from a representative experiment (upper panels). Lower panels represent the levels of MDM infection (infection index), and are means of at least 4 independent experiments performed with MDMs of 4 different donors. Error bars represent 1 SEM. Statistical significance was determined using the Anova test, and *P* values were obtained by Dunnett's post-test correction (ns, $P>0.05$).
