# Supplementary material for: HIV-1 cell-to-cell infection of macrophages escapes type I interferon and host restriction factors, and is resistant to antiretroviral drugs
Source: PLoS Pathog. 2025 Apr 28;21(4):e1013130. doi: 10.1371/journal.ppat.1013130 (PMC12064042; doi:10.1371/journal.ppat.1013130)
Supplement: S3 Fig — Jurkat T cells were infected with WT or ΔNef viruses derived from the NLAD8 or 89.6 viral strains, and then cocultured for 24 h with MDMs before elimination of T cells. (A and B) MDMs were analyzed by flow cytometry after intracellular Gag staining just after the 24 h-coculture or 6 days after elimination of T cells. Results are expressed as the percentages of Gag + MDMs relative to that determined by coculture of MDMs with Jurkat cells infected with WT NLAD8 (A) or 89.6 (B) viruses (100%). Each dot corresponds to one donor. The results represent the means of at least 4 independent experiments performed with MDMs of at least 4 different donors. Error bars represent 1 SEM. Statistical significance was determined using the One-way Anova test (ns, P > 0.05; ****P < 0.0001). (C-J) MDMs were analyzed by confocal microscopy immediately after the 24h-coculture (C-F) or 6 days after elimination of T cells (G-J) with anti-Gag (green) antibodies, phalloidin (F-actin, red), and Dapi (blue). The total number of nuclei (Dapi+) per Gag + MDM was quantified on at least 100 cells. Representative images with scale bars are shown in (C, E G and I). In D, F, H and J), results are expressed as the percentage of Gag + MGCs with 1, 2, 3, 4 or more than 4 nuclei quantified from a representative experiment (right panels). In left panels), results are expressed as the means of total nucleus number per Gag + MGC and the results represent the means of at least 4 independent experiments performed with MDMs of 4 different donors. (NI), MDMs cocultured with non-infected Jurkat cells. Error bars represent 1 SEM. Statistical significance was determined using the Anova test, and P values were obtained by Dunnett’s post-test correction (ns, (ns, P > 0.05; ***, P < 0.001; ****P < 0.0001). (PDF) [file ppat.1013130.s003.pdf]

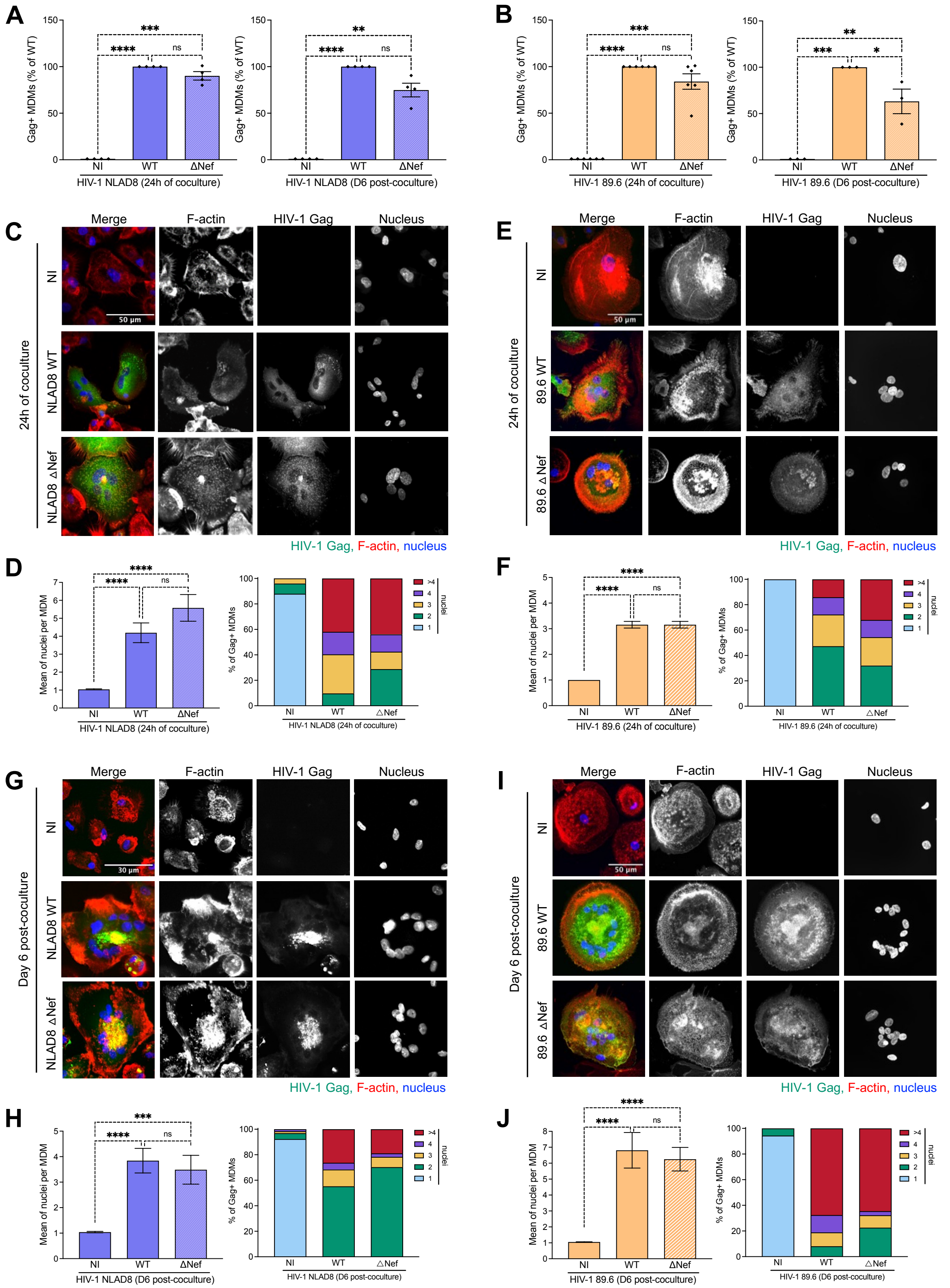

S3 Fig. Influence of Nef on HIV-1 cell-to-cell transfer to macrophages, and MGC formation. Jurkat T cells were infected with WT or ΔNef viruses derived from the NLAD8 or 89.6 viral strains, and

then cocultured for 24 h with MDMs before elimination of T cells. (A and B) MDMs were analyzed by flow cytometry after intracellular Gag staining just after the 24 h-coculture or 6 days after elimination of T cells. Results are expressed as the percentages of Gag+ MDMs relative to that determined by coculture of MDMs with Jurkat cells infected with WT NLAD8 (A) or 89.6 (B) viruses (100%). Each dot corresponds to one donor. The results represent the means of at least 4 independent experiments performed with MDMs of at least 4 different donors. Error bars represent 1 SEM. Statistical significance was determined using the One-way Anova test (ns, $P>0.05$; ****$P<0.0001$). (C-J) MDMs were analyzed by confocal microscopy immediately after the 24h-coculture (C-F) or 6 days after elimination of T cells (G-J) with anti-Gag (green) antibodies, phalloidin (F-actin, red), and Dapi (blue). The total number of nuclei (Dapi+) per Gag+ MDM was quantified on at least 100 cells. Representative images with scale bars are shown in (C, E G and I). In D, F, H and J), results are expressed as the percentage of Gag+ MGCs with 1, 2, 3, 4 or more than 4 nuclei quantified from a representative experiment (right panels). In left panels), results are expressed as the means of total nucleus number per Gag+ MGC and the results represent the means of at least 4 independent experiments performed with MDMs of 4 different donors. (NI), MDMs cocultured with non-infected Jurkat cells. Error bars represent 1 SEM. Statistical significance was determined using the Anova test, and *P* values were obtained by Dunnett's post-test correction (ns, (ns, $P>0.05$; ***, $P<0.001$; ****$P<0.0001$).

nevirapine or raltegravir) were cocultured for 6 or 24 h with NLAD8-infected Jurkat (panel B) or primary CD4 T cells (panel C), or cultured after elimination of T cells for 4 additional days in the presence of the drugs before DNA-FISH analysis. MDMs were fixed, permeabilized, stained with Dapi, and then incubated with probes for detection of the *KDM5C* gene together with the HIV-NLAD8 probe. Representative images of infected multinucleated MDMs after 6 or 24 h of coculture, or 4 days later are shown (left, middle, and right images, respectively). Scale bar is indicated.
