# Supplementary material for: HIV-1 cell-to-cell infection of macrophages escapes type I interferon and host restriction factors, and is resistant to antiretroviral drugs
Source: PLoS Pathog. 2025 Apr 28;21(4):e1013130. doi: 10.1371/journal.ppat.1013130 (PMC12064042; doi:10.1371/journal.ppat.1013130)
Supplement: S4 Fig — (A and B) Parental (mock) or SERINC5-KO Jurkat cells (clones B1, B5, and F6) were infected with WT or Nef-deleted NLAD8 (A) or 89.6 (B) viruses, and analyzed for intracellular Gag expression by flow cytometry 36 h later. Results are the means of at least 6 independent experiments performed in duplicate. (C and D) Parental (mock) or SERINC5-KO Jurkat cells infected with WT NLAD8 (C) or 89.6 (D) viruses were cocultured for 24 h with MDMs. After elimination of T cells, MDMs were analyzed immediately (24 h) or cultured for 6 additional days before analysis by flow cytometry after intracellular Gag staining. Results are the means of at least 6 independent experiments performed with MDMs from at least 6 different donors, and are expressed as the percentages of Gag + MDMs relative to those determined after coculture of MDMs with the parental infected Jurkat cells (100%). Error bars represent 1 SEM. Statistical significance was determined using the One-way Anova test (ns, P > 0.05; **, P < 0.01). (E-L) MDMs were cocultured for 24 h with parental or SERINC5-KO Jurkat cells infected with the WT viruses, and then stained just after coculture or 6 days later with anti-Gag (green) antibodies, phalloidin (F-actin, red), while the nuclei were stained with Dapi (blue), before observation by confocal microscopy. The total number of nuclei (Dapi+) per Gag + MDM was quantified on at least 100 cells Representative images are shown in E, G, I and J), and scale bars are indicated. In F, H, K and L), results are expressed as the percentage of Gag + MDMs with 2, 3, 4 or more than 4 nuclei quantified from a representative experiment (right panels). In left panels), results are expressed as the means of total nucleus number per Gag + MDM, and represent the means of at least 4 independent experiments performed with MDMs of 4 different donors. Error bars represent 1 SEM. Statistical significance was determined using the Anova test, and P values were obtained by Dunnett’s post-test correction ( [file ppat.1013130.s004.pdf]

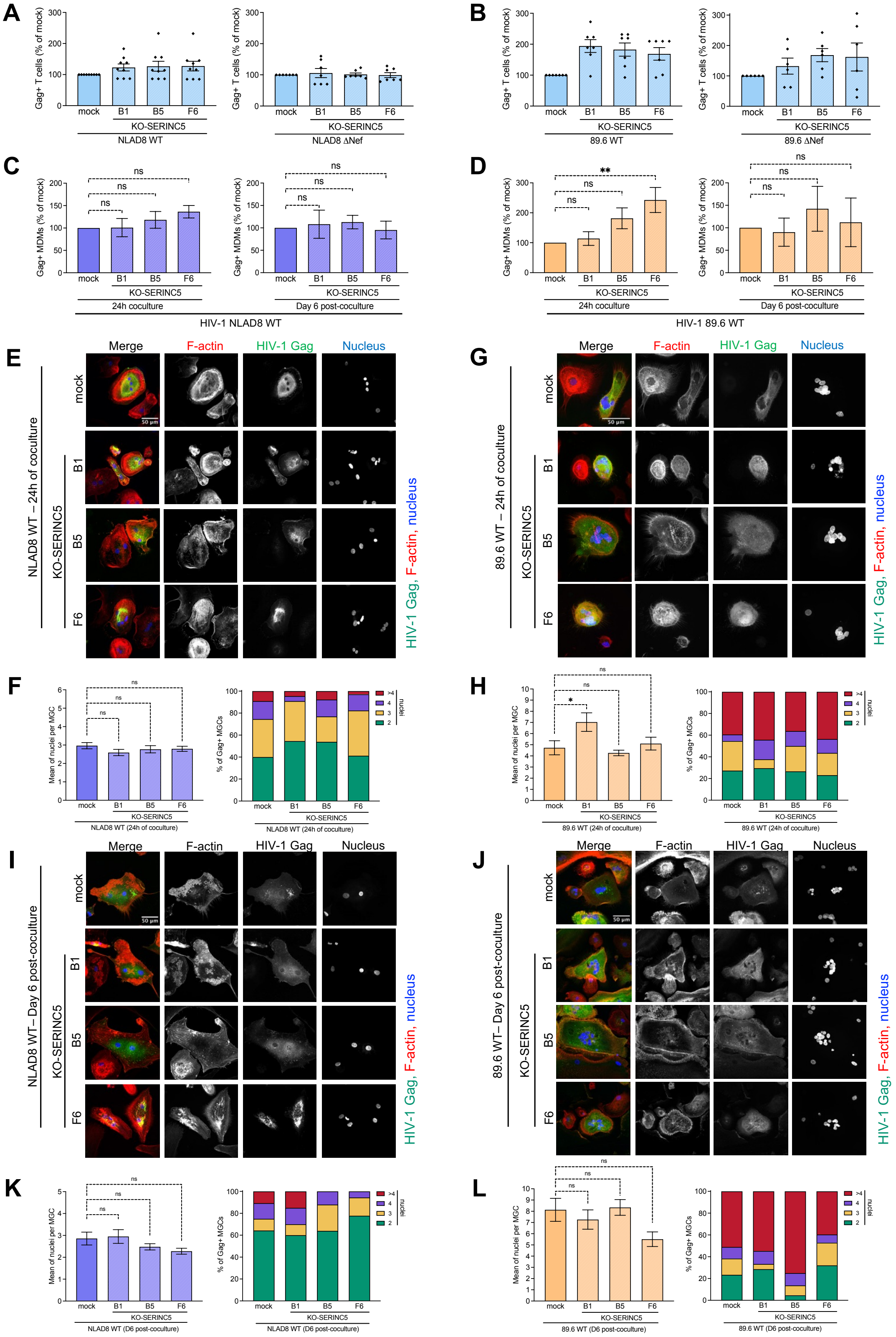

S4 Fig. Infection of MDMs by cell-cell fusion with SERINC5-deleted Jurkat cells infected with WT viruses. (A and B) Parental (mock) or SERINC5-KO Jurkat cells (clones B1, B5, and F6) were

infected with WT or Nef-deleted NLAD8 (A) or 89.6 (B) viruses, and analyzed for intracellular Gag expression by flow cytometry 36 h later. Results are the means of at least 6 independent experiments performed in duplicate. (C and D) Parental (mock) or SERINC5-KO Jurkat cells infected with WT NLAD8 (C) or 89.6 (D) viruses were cocultured for 24 h with MDMs. After elimination of T cells, MDMs were analyzed immediately (24 h) or cultured for 6 additional days before analysis by flow cytometry after intracellular Gag staining. Results are the means of at least 6 independent experiments performed with MDMs from at least 6 different donors, and are expressed as the percentages of Gag+ MDMs relative to those determined after coculture of MDMs with the parental infected Jurkat cells (100%). Error bars represent 1 SEM. Statistical significance was determined using the One-way Anova test (ns, P>0.05; **, P<0.01). (E-L) MDMs were cocultured for 24 h with parental or SERINC5-KO Jurkat cells infected with the WT viruses, and then stained just after coculture or 6 days later with anti-Gag (green) antibodies, phalloidin (F-actin, red), while the nuclei were stained with Dapi (blue), before observation by confocal microscopy. The total number of nuclei (Dapi+) per Gag+ MDM was quantified on at least 100 cells Representative images are shown in E, G, I and J), and scale bars are indicated. In F, H, K and L), results are expressed as the percentage of Gag+ MDMs with 2, 3, 4 or more than 4 nuclei quantified from a representative experiment (right panels). In left panels), results are expressed as the means of total nucleus number per Gag+ MDM, and represent the means of at least 4 independent experiments performed with MDMs of 4 different donors. Error bars represent 1 SEM. Statistical significance was determined using the Anova test, and P values were obtained by Dunnett's post-test correction (ns, P>0.05; *, P<0.05).
