# Supplementary material for: HIV-1 cell-to-cell infection of macrophages escapes type I interferon and host restriction factors, and is resistant to antiretroviral drugs
Source: PLoS Pathog. 2025 Apr 28;21(4):e1013130. doi: 10.1371/journal.ppat.1013130 (PMC12064042; doi:10.1371/journal.ppat.1013130)
Supplement: S5 Fig — MDMs were inoculated with cell-free viruses (CF), or cocultured (CTC) with autologous infected T cells for 6 h in the absence (mock) or presence of the indicated concentration of IFNa. After elimination of the virus inoculum or infected T cells, MDMs were analyzed just after the 6 h of coculture, or cultured for 6 additional days in the absence (mock) or presence of the indicated concentration of IFNa. (A and E) flow cytometry analysis. Results are expressed as the percentage of Gag + MDMs relative to that measured without IFN, after 6 h of coculture (A) or 6 days later (E). (B-D and F-H) Results represent the quantitative analysis measured from the images of cell-free (CF) infection shown in Fig 3D, lower panels. In B and F), results are expressed as the percentage of Gag + MDMs with 1, 2, 3, 4 or more than 4 nuclei. In C and G), results correspond to the level of infection (infection index) quantified from images of cell-free infection of MDMs. In D and H), results are expressed as the means of nucleus number per Gag + , and represent the means of at least 4 independent experiments performed with MDMs of 4 different donors. Error bars represent 1 SEM. Statistical significance was determined using the Anova test, and P values were obtained by Dunnett’s post-test correction (ns, P > 0.05; **, P < 0.01; ****, P < 0.0001). In I), individual staining of the representative images shown in Fig 3D. In S5J and S5K), results are expressed as the percentage of Gag + MDMs with 2, 3, 4 or more than 4 nuclei. In S5L and S5M), results correspond to the level of infection (infection index) quantified from images. (PDF) [file ppat.1013130.s005.pdf]

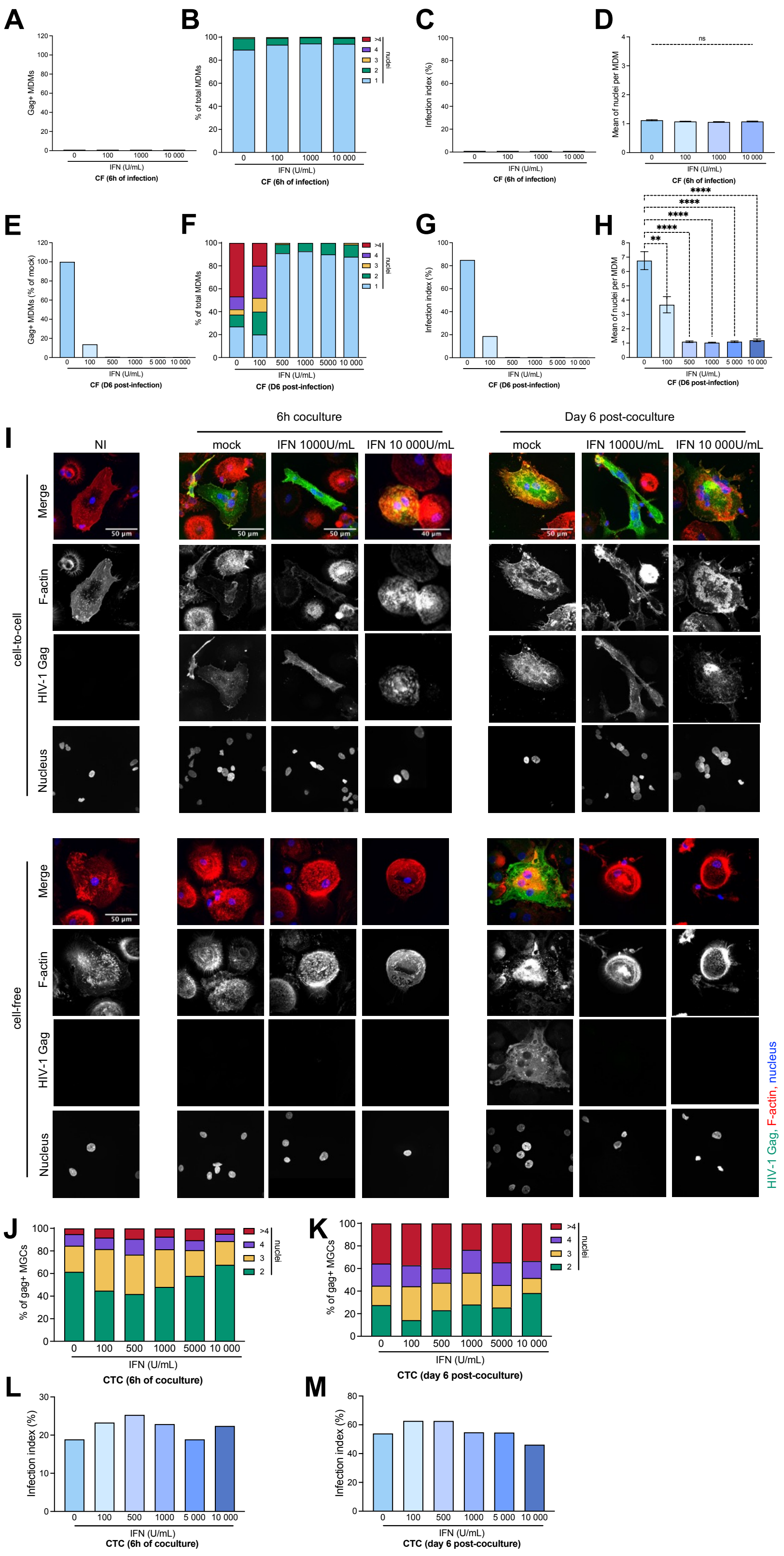

S5 Fig. IFN-I activity on HIV-1 cell-free and cell-to-cell infection of macrophages. MDMs were inoculated with cell-free viruses (CF), or cocultured (CTC) with autologous infected T cells for 6 h in the

absence (mock) or presence of the indicated concentration of IFNα. After elimination of the virus inoculum or infected T cells, MDMs were analyzed just after the 6 h of coculture, or cultured for 6 additional days in the absence (mock) or presence of the indicated concentration of IFNα. (A and E) flow cytometry analysis. Results are expressed as the percentage of Gag+ MDMs relative to that measured without IFN, after 6 h of coculture (A) or 6 days later (E). (B-D and F-H) Results represent the quantitative analysis measured from the images of cell-free (CF) infection shown in Figure 3D, lower panels. In B and F), results are expressed as the percentage of Gag+ MDMs with 1, 2, 3, 4 or more than 4 nuclei. In C and G), results correspond to the level of infection (infection index) quantified from images of cell-free infection of MDMs. In D and H), results are expressed as the means of nucleus number per Gag+, and represent the means of at least 4 independent experiments performed with MDMs of 4 different donors. Error bars represent 1 SEM. Statistical significance was determined using the Anova test, and *P* values were obtained by Dunnett's post-test correction (ns, $P>0.05$; **, $P<0.01$; ****, $P<0.0001$). In I), individual staining of the representative images shown in Figure 3D. In J and K), results are expressed as the percentage of Gag+ MDMs with 2, 3, 4 or more than 4 nuclei. In L and M), results correspond to the level of infection (infection index) quantified from images.
