# Supplementary material for: HIV-1 cell-to-cell infection of macrophages escapes type I interferon and host restriction factors, and is resistant to antiretroviral drugs
Source: PLoS Pathog. 2025 Apr 28;21(4):e1013130. doi: 10.1371/journal.ppat.1013130 (PMC12064042; doi:10.1371/journal.ppat.1013130)
Supplement: S6 Fig — (A) Experimental design, created with Biorender, for depletion of APOBEC3G with siRNA (siA3G) before treatment of MDMs with IFNα (1,000 U/mL), and virus cell-to-cell spreading from infected T cells in the presence of IFNα. After elimination of T cells, MDMs were analyzed immediately or after 6 additional days in culture by flow cytometry or confocal microscopy. In B and C) are shown the individual staining corresponding to the images shown in Fig 3N. (PDF) [file ppat.1013130.s006.pdf]

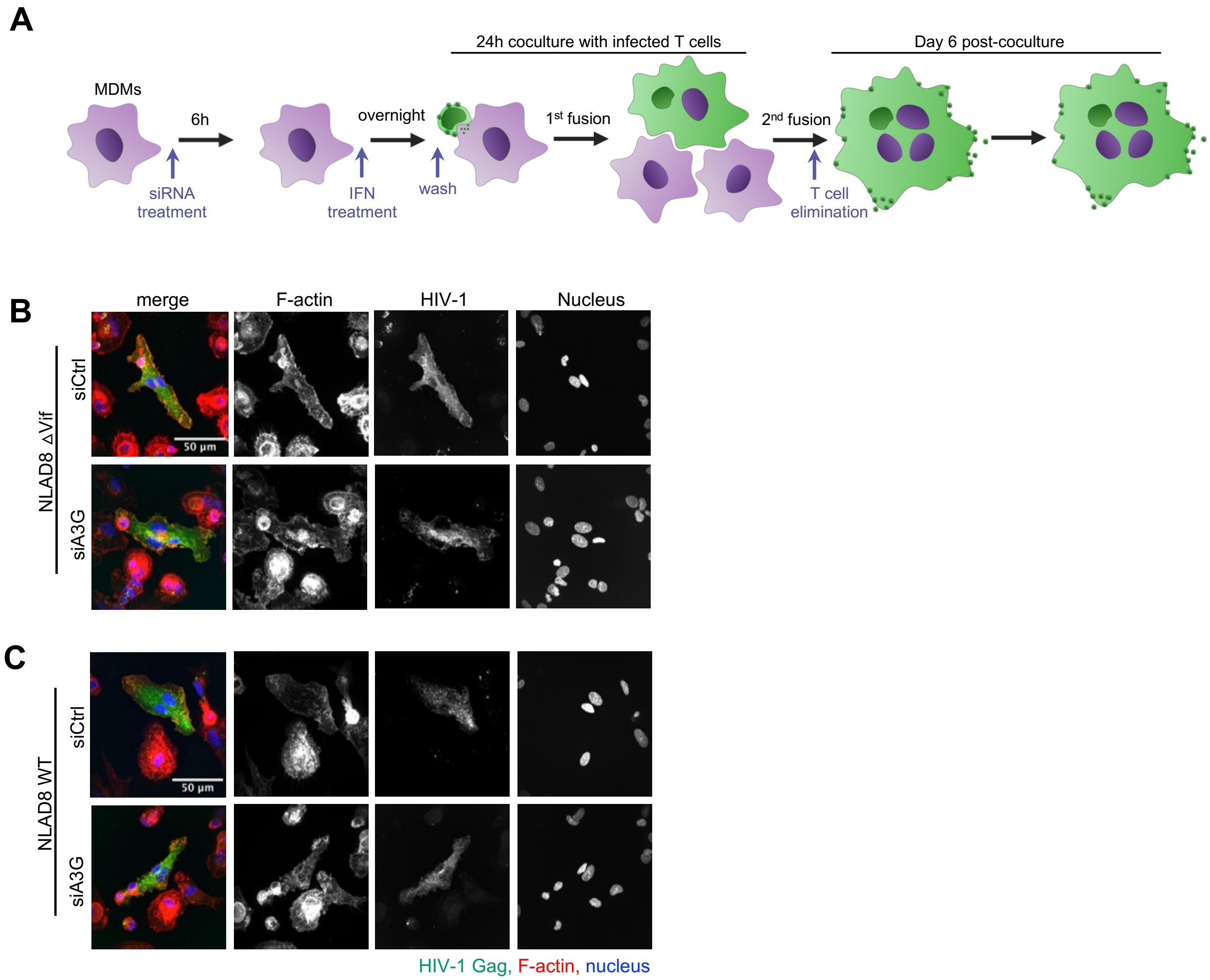

S6 Fig. Role of APOBEC3G in HIV-1 cell-to-cell infection of macrophages by cell-cell fusion with infected T cells. (A) Experimental design for depletion of APOBEC3G with siRNA (siA3G) before treatment of MDMs with IFNα (1,000 U/mL), and virus cell-to-cell spreading from infected T cells in the presence of IFNα. After elimination of T cells, MDMs were analyzed immediately or after 6 additional days in culture by flow cytometry or confocal microscopy. In B and C) are shown the individual staining corresponding to the images shown in Figure 3N.
