# Supplementary material for: HIV-1 cell-to-cell infection of macrophages escapes type I interferon and host restriction factors, and is resistant to antiretroviral drugs
Source: PLoS Pathog. 2025 Apr 28;21(4):e1013130. doi: 10.1371/journal.ppat.1013130 (PMC12064042; doi:10.1371/journal.ppat.1013130)
Supplement: S7 Fig — (A) Jurkat-LTR-GFP cells were infected with the HIV-1 NLAD8 strains, and analyzed 36 h later by flow cytometry after intracellular Gag staining. The results correspond to the percentage of Gag + T cells expressing GFP (green bar) or not (blue bar), and are the means of 4 independent experiments performed in duplicate. (B) Infected Jurkat-LTR-GFP cells were cocultured with MDMs for 24 h. After elimination of T cells, MDMs were then cultured for different period of time (from D0 to D20) before analysis by flow cytometry of the level of Gag-positive cells. The results correspond to the means of at least 4 independent experiments performed on at least 4 different donors. Error bars represent 1 SEM. (C-J) Infected Jurkat-LTR-GFP were cocultured for 24 h with MDMs. T cells were then eliminated, and the MDMs were cultured for 12 (C-F) or 20 (G-J) additional days before analysis by fluorescence microscopy after staining with anti-Gag (brown yellow) antibodies, phalloidin (F-actin, red), Dapi (Nucleus, blue) and GFP (green). Representative images are shown in C) and G), and the total number of nuclei (Dapi+) per Gag + MDM was quantified on at least 100 cells. In D and H), the results are the percentage of Gag-positive MGCs co-expressing GFP (green bar) or not (blue bar). In E and I), results are expressed as the percentage of Gag + MDMs with 1, 2, 3, or more than 3 nuclei in GFP-negative (central bar) and -positive cells (right bar). In F and J), results are the mean of nuclei per Gag + MGCs in co-expressing GFP cells (left green bar) or not (central blue bar). NI, MDMs cocultured for 24 h with noninfected Jurkat-LTR-GFP cells, and then cultured for 20 additional days before fluorescence microscopy. (PDF) [file ppat.1013130.s007.pdf]

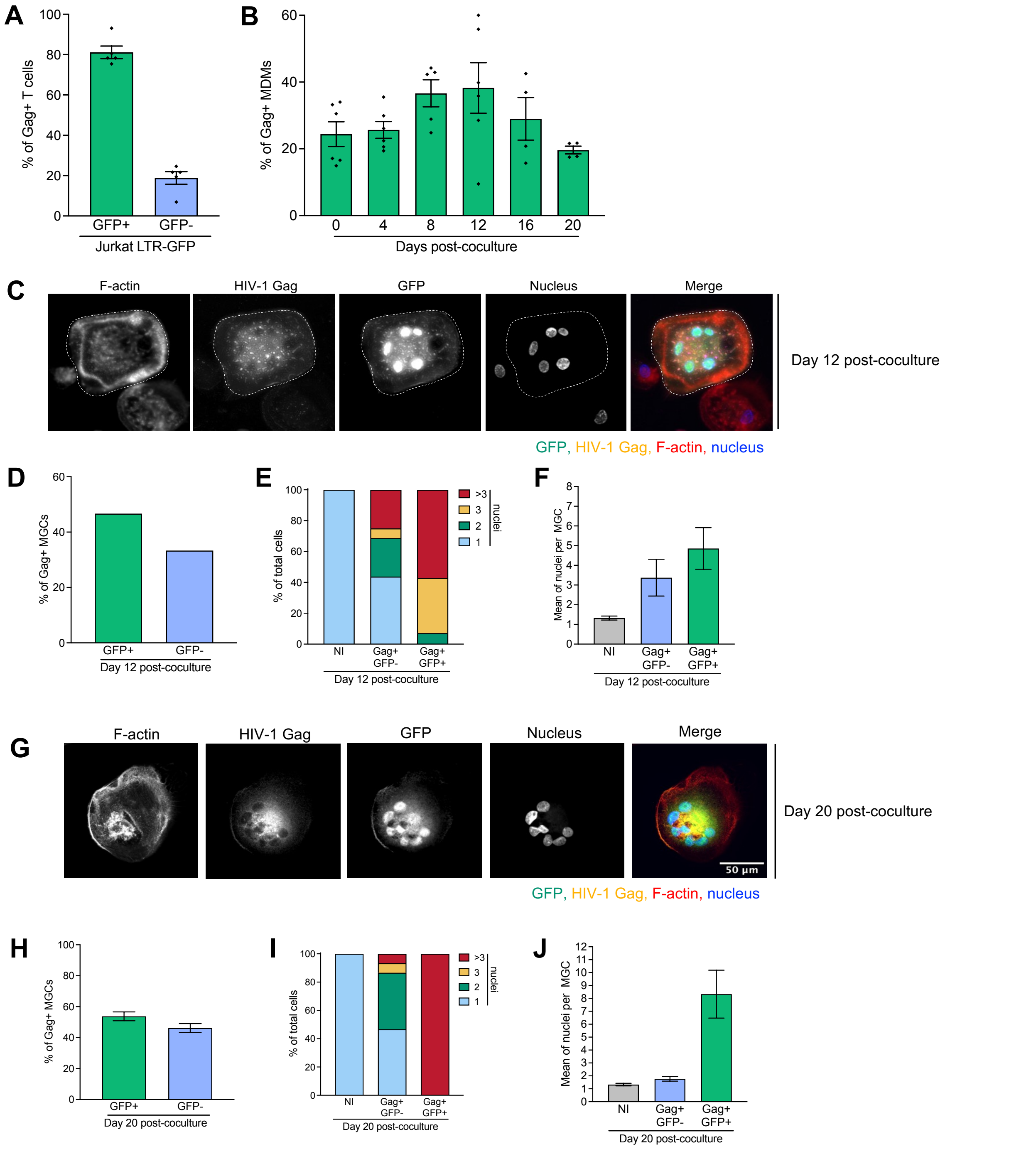

S7 Fig. Transcriptional activity of the Jurkat T cell nuclei in MGCs formed upon cell-cell fusion. (A) Jurkat-LTR-GFP cells were infected with the HIV-1 NLAD8 strains, and analyzed 36 h later by flow cytometry after intracellular Gag staining. The results correspond to the percentage of Gag+ T cells expressing GFP (green bar) or not (blue bar), and are the means of 4 independent experiments performed in duplicate. (B) Infected Jurkat-LTR-GFP cells were cocultured with MDMs for 24 h. After elimination of T cells, MDMs were then cultured for different period of time (from D0 to D20) before analysis by flow cytometry of the level of Gag-positive cells. The results correspond to the means of at least 4 independent experiments performed on at least 4 different donors. Error bars represent 1 SEM. (C-J) Infected Jurkat-LTR-GFP were cocultured for 24 h with MDMs. T cells were then eliminated, and the

MDMs were cultured for 12 (C-F) or 20 (G-J) additional days before analysis by fluorescence microscopy after staining with anti-Gag (brown yellow) antibodies, phalloidin (F-actin, red), Dapi (Nucleus, blue) and GFP (green). Representative images are shown in C) and G), and the total number of nuclei (Dapi+) per Gag+ MDM was quantified on at least 100 cells. In D and H), the results are the percentage of Gag-positive MGCs co-expressing GFP (green bar) or not (blue bar). In E and I), results are expressed as the percentage of Gag+ MDMs with 1, 2, 3, or more than 3 nuclei in GFP-negative (central bar) and -positive cells (right bar). In F and J), results are the mean of nuclei per Gag+ MGCs in co-expressing GFP cells (left green bar) or not (central blue bar). NI, MDMs cocultured for 24 h with noninfected Jurkat-LTR-GFP cells, and then cultured for 20 additional days before fluorescence microscopy.
