# Supplementary material for: HIV-1 cell-to-cell infection of macrophages escapes type I interferon and host restriction factors, and is resistant to antiretroviral drugs
Source: PLoS Pathog. 2025 Apr 28;21(4):e1013130. doi: 10.1371/journal.ppat.1013130 (PMC12064042; doi:10.1371/journal.ppat.1013130)
Supplement: S8 Fig — MDMs pretreated with the indicated antiretroviral drugs (e.i., PF74, AZT, NVP, or RAL) were infected by cell-free NLAD8 viruses or cocultured for 24 h with NLAD8-infected Jurkat cells. MDMs were then stained with anti-Gag (green) antibodies, phalloidin (F-actin, red), and Dapi (Nucleus, blue), before observation by confocal microscopy. The images shown correspond to the individual staining of the representative images shown in Fig 5B. Scale bars are indicated. (A) Images of cell-to-cell infected MDMs analyzed just after the 24 h of coculture. (B) Images of cell-to-cell infected macrophages cultured for 4 days after the coculture and elimination of infected T cells. (C) Images of cell-free infected MDMs cultured for 4 days after elimination of the viral inoculum. (D) Results are expressed as the percentage of Gag + MDMs with 1, 2, 3, 4 or more than 4 nuclei quantified from the images shown in C) on at least 100 cells. (E) Results are the means of nuclei per MDM quantified from the images shown in C), and represent the means of at least 4 independent experiments performed with MDMs of 4 different donors. Error bars represent 1 SEM. Statistical significance was determined using the Anova test, and P values were obtained by Dunnett’s post-test correction (ns, P > 0.05; ****, P < 0.0001). (PDF) [file ppat.1013130.s008.pdf]

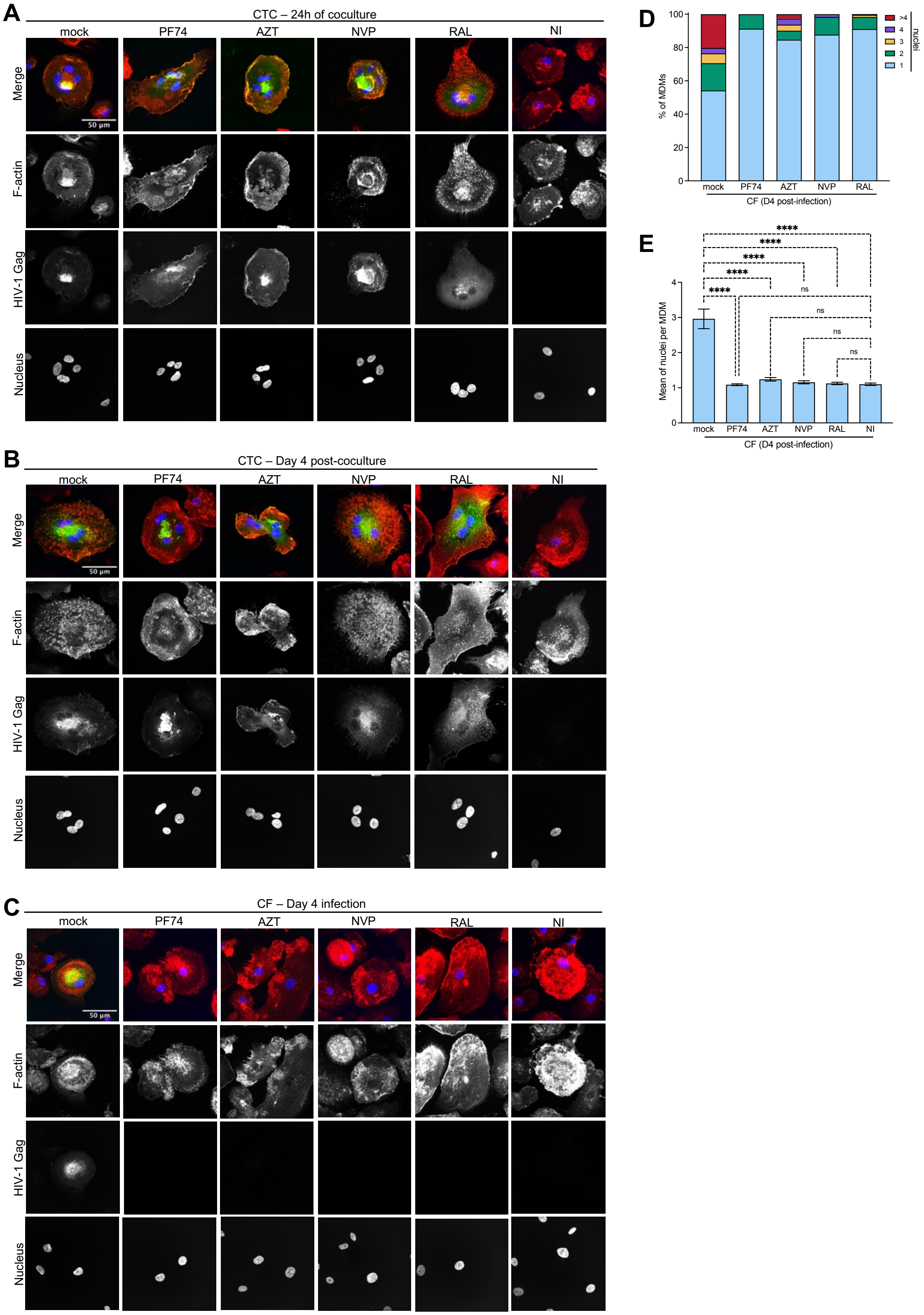

S8 Fig. Confocal microscopy images of HIV-1 cell-free and cell-to-cell infection of MDMs in the presence of antiretroviral drugs. MDMs pretreated with the indicated antiretroviral drugs (e.i., PF74,

AZT, NVP, or RAL) were infected by cell-free NLAD8 viruses or cocultured for 24 h with NLAD8-infected Jurkat cells. MDMs were then stained with anti-Gag (green) antibodies, phalloidin (F-actin, red), and Dapi (Nucleus, blue), before observation by confocal microscopy. The images shown correspond to the individual staining of the representative images shown in Fig. 5B. Scale bars are indicated. (A) Images of cell-to-cell infected MDMs analyzed just after the 24 h of coculture. (B) Images of cell-to-cell infected macrophages cultured for 4 days after the coculture and elimination of infected T cells. (C) Images of cell-free infected MDMs cultured for 4 days after elimination of the viral inoculum. (D) Results are expressed as the percentage of Gag+ MDMs with 1, 2, 3, 4 or more than 4 nuclei quantified from the images shown in C) on at least 100 cells. (E) Results are the means of nuclei per MDM quantified from the images shown in C), and represent the means of at least 4 independent experiments performed with MDMs of 4 different donors. Error bars represent 1 SEM. Statistical significance was determined using the Anova test, and *P* values were obtained by Dunnett's post-test correction (ns, $P>0.05$; ****, $P<0.0001$).
