# Supplementary material for: HIV-1 cell-to-cell infection of macrophages escapes type I interferon and host restriction factors, and is resistant to antiretroviral drugs
Source: PLoS Pathog. 2025 Apr 28;21(4):e1013130. doi: 10.1371/journal.ppat.1013130 (PMC12064042; doi:10.1371/journal.ppat.1013130)
Supplement: S9 Fig — MDMs were cocultured for 6 or 24 h with non-infected- or NLAD8-infected Jurkat or primary CD4 T cells, or cultured for 4 additional days after coculture before DNA-FISH analysis. MDMs were fixed, permeabilized, stained with Dapi, and then incubated after RNAse treatment with specific probes for detection of the KDM5C gene located on the X chromosome together with the HIV-NLAD8 probe for detection of the proviral DNA. (A) Quantification of the percentage of MGCs with T cell nuclei containing HIV-positive dots after 6 or 24 h of coculture, or 4 days later from the representative experiment shown in Fig 6. The number of HIV-positive nuclei was quantified in at least 30 MGCs. (B and C) MDMs pretreated or not with antiretroviral drugs (PF74, AZT, nevirapine or raltegravir) were cocultured for 6 or 24 h with NLAD8-infected Jurkat (panel B) or primary CD4 T cells (panel C), or cultured after elimination of T cells for 4 additional days in the presence of the drugs before DNA-FISH analysis. MDMs were fixed, permeabilized, stained with Dapi, and then incubated with probes for detection of the KDM5C gene together with the HIV-NLAD8 probe. Representative images of infected multinucleated MDMs after 6 or 24 h of coculture, or 4 days later are shown (left, middle, and right images, respectively). Scale bar is indicated. (PDF) [file ppat.1013130.s009.pdf]

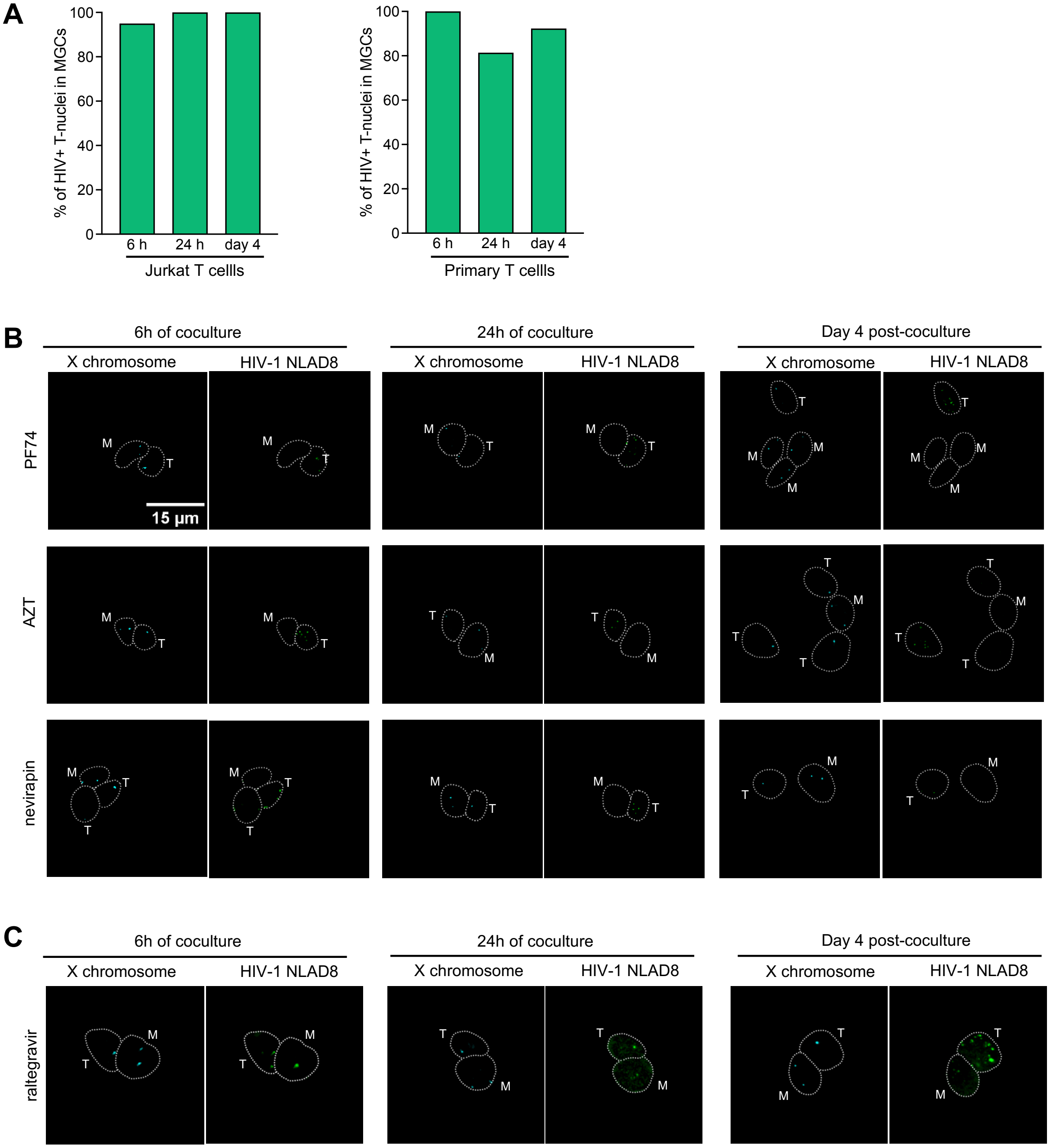

S9 Fig. FISH analysis of HIV-1 DNA in lymphoid nuclei of MGCs. MDMs were cocultured for 6 or 24 h with non-infected- or NLAD8-infected Jurkat or primary CD4 T cells, or cultured for 4 additional days after coculture before DNA-FISH analysis. MDMs were fixed, permeabilized, stained with Dapi, and then incubated after RNAse treatment with specific probes for detection of the *KDM5C* gene located on the X chromosome together with the HIV-NLAD8 probe for detection of the proviral DNA. (A) Quantification of the percentage of MGCs with T cell nuclei containing HIV-positive dots after 6 or 24 h of coculture, or 4 days later from the representative experiment shown in Fig. 6. The number of HIV-positive nuclei was quantified in at least 30 MGCs. (B and C) MDMs pretreated or not with antiretroviral drugs (PF74, AZT,

nevirapine or raltegravir) were cocultured for 6 or 24 h with NLAD8-infected Jurkat (panel B) or primary CD4 T cells (panel C), or cultured after elimination of T cells for 4 additional days in the presence of the drugs before DNA-FISH analysis. MDMs were fixed, permeabilized, stained with Dapi, and then incubated with probes for detection of the *KDM5C* gene together with the HIV-NLAD8 probe. Representative images of infected multinucleated MDMs after 6 or 24 h of coculture, or 4 days later are shown (left, middle, and right images, respectively). Scale bar is indicated.
